# Chromosome-level genome assembly of *Mentha longifolia* L. reveals gene organization underlying disease resistance and essential oil traits

**DOI:** 10.1093/g3journal/jkac112

**Published:** 2022-05-12

**Authors:** Kelly J Vining, Iovanna Pandelova, Iris Lange, Amber N Parrish, Andrew Lefors, Brent Kronmiller, Ivan Liachko, Zev Kronenberg, Narayanan Srividya, B Markus Lange

**Affiliations:** Department of Horticulture, Oregon State University, Corvallis, OR 97331, USA; Department of Horticulture, Oregon State University, Corvallis, OR 97331, USA; M.J. Murdock Metabolomics Laboratory, Institute of Biological Chemistry, Washington State University, Pullman, WA 99164-6340, USA; M.J. Murdock Metabolomics Laboratory, Institute of Biological Chemistry, Washington State University, Pullman, WA 99164-6340, USA; M.J. Murdock Metabolomics Laboratory, Institute of Biological Chemistry, Washington State University, Pullman, WA 99164-6340, USA; Center for Quantitative Life Sciences, Oregon State University, Corvallis, OR 97331, USA; Phase Genomics, Seattle, WA 98109, USA; Pacific Biosciences, Menlo Park, CA 94025, USA; M.J. Murdock Metabolomics Laboratory, Institute of Biological Chemistry, Washington State University, Pullman, WA 99164-6340, USA; M.J. Murdock Metabolomics Laboratory, Institute of Biological Chemistry, Washington State University, Pullman, WA 99164-6340, USA

**Keywords:** *Mentha longifolia*, aromatic plant, essential oil, genome, mint, pulegone reductase, *Verticillium*

## Abstract

*Mentha longifolia* (L.) Huds., a wild, diploid mint species, has been developed as a model for mint genetic and genomic research to aid breeding efforts that target Verticillium wilt disease resistance and essential oil monoterpene composition. Here, we present a near-complete, chromosome-scale mint genome assembly for *M. longifolia* USDA accession CMEN 585. This new assembly is an update of a previously published genome draft, with dramatic improvements. A total of 42,107 protein-coding genes were annotated and placed on 12 chromosomal scaffolds. One hundred fifty-three genes contained conserved sequence domains consistent with nucleotide binding site-leucine-rich-repeat plant disease resistance genes. Homologs of genes implicated in Verticillium wilt resistance in other plant species were also identified. Multiple paralogs of genes putatively involved in *p*-menthane monoterpenoid biosynthesis were identified and several cases of gene clustering documented. Heterologous expression of candidate genes, purification of recombinant target proteins, and subsequent enzyme assays allowed us to identify the genes underlying the pathway that leads to the most abundant monoterpenoid volatiles. The bioinformatic and functional analyses presented here are laying the groundwork for using marker-assisted selection in improving disease resistance and essential oil traits in mints.

## Introduction

Mints (*Mentha* spp.) are important specialty crops, grown mostly in temperate climates for distilled essential oils and as aromatic herbs (Lawrence 2007). Commercial cultivars are clonally propagated polyploids, originating from a series of natural hybridizations, which presents difficulties for both breeding and genetic studies. Spearmint (*M. spicata* L.) may be either triploid (2n = 3x = 36) or tetraploid (2n = 4x = 48), with variable fertility. *Mentha* *spicata* is believed to be derived from hybridization between *M. suaveolens* Ehrh. and *M. longifolia* (L.) Huds. Peppermint (*M.* × *piperita* L.) is a sterile hexaploid (2n = 6x = 72) with high susceptibility to *Verticillium* wilt, a disease caused by the fungus *Verticillium dahliae*. “Black Mitcham,” the commercially most valuable mint in temperate climates, is believed to have arisen from hybridization between *M. aquatica* L. (2n = 8x = 96) and *M. spicata* (2n = 4x = 48) (Tucker 2012). *Mentha* *longifolia*, commonly called “horsemint,” is a wild, diploid (2n = 2x = 24) mint species with notable phenotypic diversity, and is represented at the USDA National Clonal Germplasm Repository by accessions from numerous geographical locations (Chambers and Hummer 1994). The species has been developed as a model for *Mentha* genetic studies (Vining *et al.* 2005, 2007) and was the source of DNA for the first *Mentha* draft genome from wilt-resistant accession CMEN 585 (Vining *et al.* 2017).

A central goal of mint breeding efforts has been to develop cultivars with both peppermint-type oil properties and resistance to *Verticillium* wilt. One approach taken in the 1950s by the A.M. Todd company built on gamma irradiation of Black Mitcham peppermint, followed by field evaluation of 100,000 irradiated individuals for wilt resistance (Murray and Todd 1972). These activities resulted in the release of 2 cultivars considered to have stronger wilt resistance relative to Black Mitcham: ‘Todd’s Mitcham’ and ‘Murray Mitcham’ (Murray and Todd 1972; Todd *et al.* 1977). A second approach involved the “resynthesis” of *M.* × *piperita* from parental species. Controlled crosses between accessions of *M. aquatica* and *M. spicata* resulted in F_1_ progeny with a range of chromosome complements, morphological phenotypes and oil compositions (Murray *et al.* 1972). Irradiation-based breeding relied on the labor-intensive evaluation of large populations and controlled crosses were subject to transgressive segregation and aberrant chromosome segregation, resulting in unpredictable phenotypes (Tucker 2012). In summary, mint breeding has thus far suffered from a lack of knowledge of the genome context and regulatory mechanisms of the genes underlying complex traits. The chromosome-level assembly of a mint reference genome reported here directly addresses some of these limitations.

Relative to fragmented draft assemblies, chromosome-level genome assemblies enable vastly improved gene annotation, particularly for expansive gene families such as monterpene synthases, which determine mint essential oil composition. A second category of genes relevant to mint breeding for *Verticillium* wilt resistance encompasses disease resistance genes (“R” genes), a well-studied feature of plant genomes. R genes typically occur in complex, multigene clusters on most or all chromosomes (Jupe *et al.* 2012; Marone *et al.* 2013; Sekhwal *et al.* 2015; Neupane *et al.* 2018). R gene clusters consist of both apparently functional genes (based on predicted protein-coding sequence), and nonfunctional pseudogenes that are considered evolutionary remnants. These clusters are thought to have arisen by segmental and tandem duplication events, and to evolve relatively rapidly (Michelmore and Meyers 1998; Meyers *et al.* 2003; McHale *et al.* 2006).

The largest category of R genes encode N-terminal Nucleotide Binding Site (NBS) and C-terminal Leucine-Rich Repeat (LRR) functional domains (referred to as NLR when both domains are present) (McHale *et al.* 2006). The NBS domain consists of ∼300 amino acids, and contains 8 characteristic conserved motifs: P-loop (Kinase-1a), Kinase-2, RNBS-A, RNBS-B, RNBS-C, RNBS-D, GLPL, and MHDV (Meyers *et al.* 1999, 2003). The LRR domain is hypervariable, and may interact directly or indirectly with pathogen effector proteins (DeYoung and Innes 2006). The large NLR gene family can be divided into Toll-interleukin 1 receptor-like (TNL) or nTNL subfamilies based on the presence or absence of an N-terminal Toll/Interleukin-1 Receptor-like (TIR) domain in the encoded proteins (McHale *et al.* 2006). Since many nTNL genes encode N-terminal Coiled-Coil (CC) domains, nTNL genes are often referred to as CNLs (Meyers *et al.* 2003). In addition to the TNL or CNL subfamilies, a small number of nTNLs have an N-terminal domain with homology to that of RPW8 (*RESISTANCE TO POWDERY MILDEW 8*) (Zhong and Cheng 2016).

The first major R gene implicated in *Verticillium* wilt resistance, “*Ve1*,” was found in tomato (*Solanum lycopersicum*) (Kawchuk *et al.* 2001). Since that initial discovery, *Ve1* homologs have been identified in a variety of dicotyledonous plants, including other *Solanum* species (Song *et al.* 2017), and in genera *Gossypium* (Chen *et al.* 2016) and *Mentha* (Vining and Davis 2009). Other studies have elucidated details of tomato Ve1 protein function, including positive regulation of Ve1 by NRC1, ACIF, MEK2, and SERK3; Ve1 interactions with downstream signaling proteins EDS1 and NDR1; and interactions with fungal avirulence proteins (Fradin *et al.* 2009, 2014; de Jonge *et al.* 2012; Zhang *et al.* 2013). One goal of the present study is to produce a complete mint R gene annotation, including chromosomal locations of R gene clusters, and potential homologs of genes in the *Ve1* signaling pathway.

**Fig. 1.**
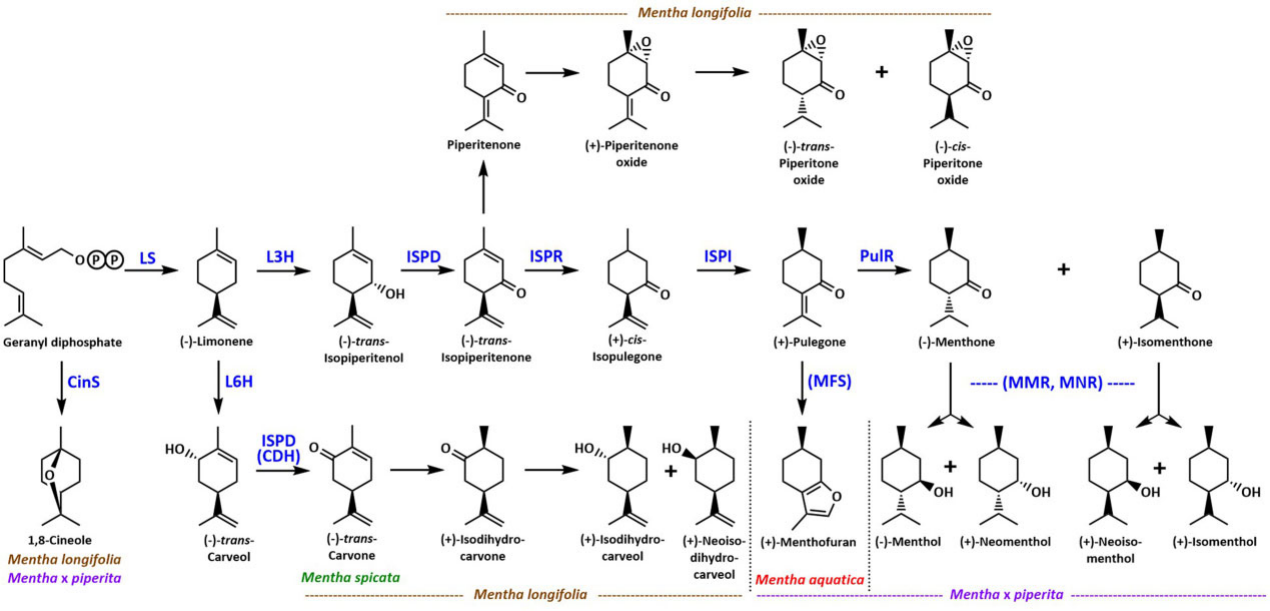
Outline of *p*-menthane monoterpene biosynthesis in mint. LS, (−)-Limonene synthase; CinS, 1,8-Cineole synthase; L3H, (−)-Limonene-3-hydroxylase; L3H, (−)-Limonene-6-hydroxylase; ISPD(CDH), (−)-Isopiperitenone dehydrogenase (Carveol dehydrogenase); ISPR, (−)-Isopiperitenone reductase; MFS, (+)-Menthufuran synthase; PulR, (+)-Pulegone reductase; MMR, (−)-Menthone:( −)-menthol reductase; MNR, (−)-Menthone:(+)-neomenthol reductase.

The biochemistry and molecular genetics of essential oil constituents in mint have been established over the last 2 decades, and the most pressing questions today revolve around the genomic organization and regulation of oil biosynthetic pathways. Monoterpenoids of the *p*-menthane class are particularly abundant in the genus *Mentha* (Lange 2015) (Fig. 1). The *p*-menthane monoterpene-specific enzymatic reactions are initiated by (−)-limonene synthase (LS) (Colby *et al.* 1993). Oxygenation can then occur at C3 [catalyzed by (−)-limonene 3-hydroxylase; L3H] or C6 [catalyzed by (−)-limonene 6-hydroxylase; L6H] (Lupien *et al.* 1999). Oxidation of the hydroxyl group at C3 or C6, depending on the availability of the appropriately hydroxylated substrate, occurs through catalysis by the multifunctional (−)-*tran*s-isopiperitenol/(−)-*trans*-carveol dehydrogenase (ISPD) to yield either (−)-*tran*s-isopiperitenone [C3 functionalization; prevalent in *Mentha* × *piperita* L. (“Black Mitcham” peppermint)] or (−)-*trans*-carvone [C6 functionalization; prevalent in *M.* *spicata* L. (“Native” spearmint)] (Ringer *et al.* 2005) (Fig. 1). The C3-functionalized intermediate (−)-*trans*-isopiperitenone can be converted to piperitenone and piperitone oxide via an as-yet unknown sequence of reactions; alternatively, this intermediate can also undergo consecutive reduction and isomerization reactions, catalyzed by (−)-*trans*-isopiperitenone reductase (ISPR) (Ringer *et al.* 2003) and (+)-*cis*-isopulegone isomerase (ISPI), to form (+)-pulegone, another branch-point intermediate (Croteau and Venkatachalam 1986). Cyclization of (+)-pulegone to (+)-menthofuran [catalyzed by (+)-menthofuran synthase; MFS; Bertea *et al.* (2001)] is prevalent in *Mentha aquatica* L. (watermint) (Vining *et al.* 2019), while a series of reductions [catalyzed by (+)-pulegone reductase (PulR), (−)-menthone:( −)-menthol reductase (MMR) and (−)-menthone:(+)-neomenthol reductase (MNR)] generates monoterpenoids typically accumulated in peppermint oil (Ringer *et al.* 2003; Davis *et al.* 2005). A preliminary analysis of distillates indicated that different *M. longifolia* accessions can contain vastly different primary oil constituents (Vining *et al.* 2007), and it was therefore an important goal of the present study to assess the correlation of gene content and oil profile in CMEN 585.

In order to build a foundation for mint genomics and marker-assisted breeding, we recently completed a draft reference genome sequence alignment for diploid *M. longifolia* (Vining *et al.* 2017). This draft assembly, while instrumental in providing a first glimpse at genome organization and serving as blueprint for the development of molecular markers, did not represent fully contiguous chromosomes. Here, we report on an updated reference genome for *M. longifolia* accession CMEN 585 with greatly improved gene annotation in chromosomal context. An important focus of the present study was to catalog the number, composition, and chromosomal locations of R gene clusters and monoterpenoid biosynthetic genes. These studies were complemented by the functional characterization of the genes underlying the monoterpenoid pathway, thereby enabling the generation of genomics-based resources for mint varietal improvement.

## Materials and methods

### Sequencing and contig scaffolding

High molecular weight genomic DNA was isolated from young, unexpanded leaf tissue of *M. longifolia* L. (accession CMEN 585, obtained from the USDA National Clonal Germplasm Repository in Corvallis, OR, USA) using a modified cetyl trimethyl ammonium bromide method (Healey *et al.* 2014). The sequencing sample was prepared using 20−40 µg DNA and loaded into 16 SMRT cells on a PacBio Sequel instrument at the Oregon State University Center for Genome Research and Biocomputing. The resulting sequence data were assembled using Falcon and HGAP4 (Chin *et al.* 2016) within the SMRT Link 3.1.1 software package (Pacific Biosciences, Menlo Park, CA, USA). This assembly, designated “v2.0,” consisted of 1,243 scaffolds with an N50 of just over 1 megabase (Mb) (Table 1). The v2.0 scaffolds were error-corrected with Illumina HiSeq3000 data (∼35× genome coverage). Illumina reads were aligned to the PacBio contigs using the BWA software package version 0.7.12-r1039 (seed length setting of 31). The alignment was then used for error correction with Pilon version 1.22 (run with default parameters, with the exception that “mindepth” was set to 0.5) (Li and Durbin 2009; Walker *et al.* 2014).

**Table 1.** Genome assembly metrics for *M. longifolia* genome assembly versions 1.0–3.0.

Metric	Mlong585_v1.0	Mlong585_v2.0	Mlong585_v3.0
No. scaffolds	89,872	2,256	326
Longest scaffold (bp)	65,819	5,978,549	46,699,537
Scaffold N50 (bp)	4,474	517,433	37,537,474
Total assembly size (Mbp)	353,287,234	470,450,732	469,158,238

The error-corrected Mlong585_v2.0 contigs were used for chromosome scaffolding with chromosome conformation capture (Hi-C) methods as follows. Tissue processing, chromatin isolation, library preparation, and 150-bp PE sequencing were performed by Phase Genomics (Seattle, WA, USA). PE reads were aligned to the polished contigs using the Burrows-Wheeler Aligner (BWA mem) (Li and Durbin 2009, 2010). Contigs were clustered, ordered, and oriented using Proximo, an adapted proximity-guided assembly platform based on the LACHESIS method (Burton *et al.* 2013; Bickhart *et al.* 2017) with proprietary parameters developed at Phase Genomics as described by Peichel *et al.* (2017). In brief, Proximo aligned the Hi-C PE reads to the contigs, and the number of pairs linking contigs was used to cluster scaffolds into chromosomal groups using a hierarchical clustering algorithm, where the final number of groups was specified as the number of the haploid chromosomes. Proximo then ordered the scaffolds based on Hi-C link densities, with the expectation that closely linked scaffolds will have higher link densities. Lastly, the orientation of ordered contigs within chromosomal groups was determined using a weighted directed acyclic graph of all possible orientations based on the exact locations of the Hi-C links between scaffolds. Gaps between scaffolds within this assembly were N-filled with 100 Ns. The scaffolds were manually curated using the Juicebox Assembly Tools module (Durand *et al.* 2016). This process resulted in a final set of genome scaffolds designated “v3.0.”

### Linkage map construction

As an additional check of the v3 genome assembly, a genetic linkage map was constructed using genotyping-by-sequencing (GBS) data from a *Mentha longifiolia* F2 population. This population was generated by crossing CMEN 585 with CMEN 584, another *M. longifolia* accession from the USDA National Clonal Germplasm Repository that, like CMEN 585, originated from South Africa. The resulting F1 population was screened for *Verticillium* wilt resistance and essential oil profile, and a wilt-resistant F1 individual was selected and self-pollinated to produce the 94-member “South African F2” population, abbreviated SAF2.

GBS data were generated using restriction enzyme ApeKI, followed by adapter ligation and sequencing (2×101 bp). The data were aligned to the *M. longifolia* v3.0 assembly using bwa v. 0.7.17-r1188. The Stacks2 pipeline was used to process the aligned GBS data and call single nucleotide polymorphisms (SNPs) (Catchen *et al.* 2011, 2013). The final set contained 5,882 markers that were called in at least 75% of the population and had a minimum minor allele frequency of 5%. The marker positions SNPs in the reference genome were anonymized (all coded as “chromosome 1” with no basepair position) prior to linkage mapping.

The linkage map was constructed using the R package “qtl.” SAF2 individuals with fewer than 900 markers were excluded from the analysis, leaving a total of 89 individuals. SNP markers were retained if they were genotyped in at least 70 SAF2 individuals. After filtering for duplicate markers and those with high segregation distortion, 1,701 markers were used to form linkage groups (LGs) with a maximum pairwise marker recombination frequency of 0.35 and a minimum LOD score of 9. Individual pairs of markers were manually inspected, and alternate orders of markers on LGs were compared with minimize numbers of obligate crossovers and sizes of gaps. The final LGs’ markers were compared with their positions in the genome assembly to look for misassemblies.

### Genome annotation

Gene annotation was performed using the chromosome-scaffolded *M. longifolia* v3.0 genome assembly. Repetitive sequence elements were masked prior to gene annotation. A custom *M. longifolia* repeat library was created using methods described in Campbell *et al.* (2014) with additional custom Perl scripts. The RepeatMasker package was employed to mask the genome assembly using the custom repeat database (Tarailo‐Graovac and Chen 2009), while LTRharvest and LTRdigest served to identify and classify LTR retrotransposons (Ellinghaus *et al.* 2008; Steinbiss *et al.* 2009). The MAKER-P pipeline was used to annotate genes in the v3.0 assembly in an iterative process. Both transcriptome data sets and protein data sets were used for homology-based gene prediction. Pooled transcriptome data sets from glandular trichomes *M. aquatica*, *M.* × *piperita*, and *M. spicata* (NCBI short read archive accession numbers SRX533474, SRX53346, and SRX533472) were used as transcript evidence. Protein evidence consisted of 587 proteins from 8 *Mentha* species (*M. aquatica*, *M. arvensis, M. canadensis*, *M. longifolia*, *M. spicata*, *M.* x*piperita*, *M. pulegium*, *M. spicata*, and *M. suaveolens*) downloaded from GenBank. In addition to homology-based prediction, ab initio gene prediction was done using both SNAP and Augustus. All gene models from the *M. longifolia* CMEN 585 V1 genome annotation (Vining *et al.* 2017) were used to train the SNAP ab initio gene predictor (Korf 2004). *Arabidopsis thaliana* was used as the Augustus gene prediction species model. The resulting set of 76,060 predicted genes was filtered to remove short (encoding <50 amino acids) open reading frames with low support (Annotation Edit Distance >0.5), resulting in a final tally of 42,107 genes. This gene set was subject to functional prediction analysis with Blast2GO version 5 (Conesa *et al.* 2005). Assembly completeness was assessed with the Benchmarking Universal Single-Copy Orthologs (BUSCO) pipeline version 3 (Simão *et al.* 2015), searching with the Embryophyta dataset of 1,440 single-copy conserved genes. Both RepeatMasker and TRF version 4.09 (Benson 1999) were used to analyze repetitive sequence elements, including long tandem repeats indicative of centromeric DNA. To identify potential centromeric repeats, the TRF raw output was filtered to exclude motif lengths <100 bp with occurrence of <20 tandem copies. Putative centromeric repeat motifs were from each chromosome were aligned to each other and compared with those published previously (Melters *et al.* 2013). Sequence and annotation files were integrated using the Web Apollo platform (Lee *et al.* 2013), and the annotated assembly is publicly accessible via the Mint Genome Resource (http://langelabtools.wsu.edu/mgr/).

To identify potential disease resistance gene homologs, 42,107 predicted protein-coding genes of *M. longifolia* were searched against the PRGdb database version 3.0 using the DRAGO2 tool (Osuna-Cruz *et al.* 2018). The NBS regions were extracted from the 153 *M. longifolia* protein sequences predicted to have both NBS and LRR domains; these were used to generate a multiple sequence alignment with CLUSTALW. A phylogenetic tree of these sequences was then produced using the Maximum Likelihood method in MEGA X, with a total of 3,081 positions in the final dataset (Kumar *et al.* 2018). MEME version 5.05 (Bailey *et al.* 2009) was employed to discover subdomains conserved among these putative NBS sequences. To identify potential homologs of genes implicated in *Verticillium* wilt resistance, BLASTp searches were conducted using the predicted amino sequences of 6 genes with well-studied roles in early recognition and resistance response signaling in tomato (Fradin *et al.* 2009) (Table 5).

Genes involved in monoterpenoid biosynthesis belong to larger gene families. To identify candidate genes for the monoterpenoid biosynthetic pathway in *M. longifolia*, sequences of functionally characterized genes from other members of the mint family (*M. spicata* and *M. × piperita*) were used in BLASTn searches against the *M. longifolia* CMEN 585 genome v3.0 assembly. The functional characterization of the *M. longifolia* candidate genes is described below. Blast results were correlated with predicted gene models as well as transcriptome and functional annotation data. Intron/exon boundaries of essential oil genes, as assigned by the MAKER-P platform (Campbell *et al.* 2014), were assessed manually by aligning genomic DNA and transcript sequence data.

### Greenhouse growth, harvest, and processing of plants for essential oil analysis

Plants were maintained under greenhouse growth conditions as described before (Lange *et al*., 2011). The harvest of leaf material, subsequent hydrodistillation, and analysis of the recovered essential oil by gas chromatography were performed as described in Vining *et al.* (2019).

### Gene cloning, heterologous expression, and recombinant protein purification

Candidate genes were PCR-amplified and inserted into the pET28b vector (Novagen) were transformed into chemically competent *E. coli* BL21(DE3) cells and plated onto LB-agar plates containing 50 µg/ml kanamycin. After incubation at 37°C for 16 h, single colonies were picked into a glass vial with 5 ml LB medium containing 50 µg/ml kanamycin. These starter cultures were shaken (200 rpm) at 37°C for 8 h and then transferred to a 500-ml Erlenmeyer flask containing 150 ml LB medium (containing 50 µg/ml kanamycin). Cultures were incubated at 37°C for roughly 16 h until an optical density of 0.9–1.0 (at 600 nm) was reached. The flask was cooled to 16°C and isopropyl β-d-1-thiogalactopyranoside added to a final concentration of 0.5 mM. Cultures were incubated at 16°C (200 rpm) for 24 h. Cells were precipitated by centrifugation at 5,000×*g* at 4°C for 10 min. The supernatant was carefully decanted and the remaining cell pellet resuspended in 500 µl 3-morpholino-2-hydroxypropanesulfonic acid buffer (pH 7.5). Cells were lysed by ultrasonication (3 pulses of 20 s each, cooling for 60 s on ice between pulses) and cell fragments precipitated by centrifugation at 13,000×*g* at 4°C for 30 min. The supernatant containing soluble proteins was loaded onto nickel affinity columns and the purification of His-tagged target proteins performed according to the manufacturer’s instructions (Bio-Rad). Aliquots of crude and processed extracts were examined by SDS-PAGE to assess the progress of the purification. Fractions with a target protein purity of >90% [comparison of band intensities following staining with Colloidal Blue (ThermoFisher)] were further processed by removing smaller molecules using a P6 column according to the manufacturer’s instructions (Bio-Rad). Bradford assays were used to quantify the purified, recombinant protein used in enzyme assays.

### Enzyme assays

Enzyme assays were carried out using previously published protocols (Ringer *et al.* 2003, 2005; Davis *et al.* 2005). Substrates and products for the assays were part of our chemical library. Kinetic assays of PulR were performed with 25 µg of purified, recombinant protein in phosphate-citrate buffer (pH 6), in the presence of 1 mM NADPH and with varying substrate concentration. The total volume of the assay was 200 µl. To speed up the detection of substrates and products, conditions for GC-FID (6890N, Agilent Technologies) using a DB-WAX column (60 m × 0.25 mm × 0.25 µm; J&W Scientific) were modified as follows: front inlet and detector temperature 270°C, inlet mode splitless, injection volume 1 µl, carrier gas flow (He) 0.9 ml/min; initial oven temperature 85°C (hold for 4 min), then linear gradient to 130°C at 30°C/min (hold for 3 min), and a second linear gradient to 235°C at 20°C/min (hold for 10 min). Quantitation was achieved in the ChemStation B.03.02 software (Agilent Technologies) based upon calibration curves with known amounts of authentic standards and normalization to the sample weight and peak area of the internal standard (camphor). Prism 8 software (GraphPad) was used to calculate kinetic values from assay data.

### Transcript abundance

A publicly available dataset, reflecting genes expressed in isolated *M. longifolia* CMEN 585 glandular trichomes (NCBI Short Read Archive accession number SRX 1818411), was analyzed to assess the abundance of transcripts related to essential oil biosynthesis. Sequence reads were assembled with Trinity (Grabherr *et al*. 2011) and expression levels calculated using the RSEM (Li and Dewey 2011) and Bowtie (Langmead *et al*. 2009) software packages (expressed as Transcript Per Kilobase Million or TPM). Multimapped reads were counted once for each alignment and randomly assigned to the best alignment, as suggested by Deschamps-Francoeur *et al*. (2019).

## Results

### Genome sequence assembly and annotation

Long-read sequencing (“PacBio”) followed by chromosome conformation capture (“Hi-C”) methods were employed to improve the assembly of the *Mentha* reference genome. A total of 5,485,340 PacBio reads were generated, with average subread length being 8,240, and longest subread 112,019 bp, overall yielding 45 Gb of sequence data (94x genome coverage). The assembly generated from these reads, “Mlong585_v2.0,” spanned 470,450,732 bp, and contained 2,256 contigs with an N50 517,433 (Table 1). The v2.0 assembly was used as the input assembly to the chromosome scaffolding process. The resulting “Mlong585_v3.0” genome assembly consisted of 12 pseudochromosomes plus 314 unanchored scaffolds, for a total of 326. The v3.0 assembly size was about 1.3 Mb smaller than the v2.0 assembly but was still within the expected range based on flow cytometry estimates. The v3.0 scaffold N50 was greatly increased to more than 37 Mb. The 12 chromosomal scaffolds ranged in length from 46,699,537 bp to 29,660,084 bp (Table 2). There were 3,266 gaps of 100 Ns remaining after scaffolding; these accounted for <1% of their cumulative length. Alignment of the v1.0 scaffolds to the v3.0 scaffolds showed the high degree of fragmentation of the first draft genome: only ∼51% of the v3.0 genome was covered by v1.0, and the longest alignment between any v1.0 and v3.0 scaffold pair was only 34 kb (kilobase) (Supplementary Table 1).

**Table 2.** Chromosomal scaffold lengths and approximate centromeric repeat delimitations in the *M. longifolia* v3.0 genome assembly.

Chr	Chr length (bp)	No. *N* gaps	% *N* bp	Centromere start (bp)^a^	Centromere end (bp)	Centromere length (bp)	CenLen%^b^
1	46,699,537	290	0.062	34,489,422	36,508,933	2,019,511	4.3
2	45,526,029	342	0.075	24,622,375	25,235,431	613,056	1.3
3	45,460,755	323	0.071	35,843,645	37,229,009	1,385,364	3.0
4	44,284,954	293	0.066	20,712,891	22,388,150	1,675,259	3.8
5	43,231,337	286	0.066	31,983,996	32,181,686	197,690	0.5
6	37,537,474	208	0.055	28,695,852	29,446,671	750,819	2.0
7	36,621,300	257	0.070	24,207,106	24,865,484	658,378	1.8
8	36,842,082	281	0.076	25,371,840	26,027,713	655,873	1.8
9	33,738,042	199	0.059	16,594,927	18,347,359	1,752,432	5.2
10	33,088,173	293	0.089	8,356,615	9,717,557	1,360,942	4.1
11	29,943,784	180	0.060	8,606,149	10,200,391	1,594,242	5.3
12	29,660,084	314	0.106	1,124,031	1,806,597	682,566	2.3
					Total	13,346,132	

a Centromere start and end positions were approximated based on tandem repeat analysis.

b Centromere percentage of total chromosome length is shown.

Genome repeat identification and masking was performed prior to protein-coding gene annotation. Approximately 2% of the *M. longifolia* genome consisted of Long Interspersed Repeats (LINEs), 5% of Long Terminal Repeats (LTRs), and 35% of other transposon-like elements (Supplementary Table 2). Simple Sequence Repeats (SSRs) constituted 2% of the genome. Centromeric repeats, determined by their motif lengths of ∼150 bp, composed 0.5–5.3% of the base pairs in chromosomes, 2.8% of the total genome assembly (Table 3). The canonical centromeric repeat motif in the *M. longifolia* CMEN 585 genome was 156 bp (Supplementary Fig. 1). While the length of this motif was similar to that reported for other angiosperm taxa, the *M. longifolia* repeats could not be meaningfully aligned to published centromeric repeat sequences from 282 plant taxa (Melters *et al.* 2013), and blast searches omitting repeat masking produced no hits. The *M. longifolia* centromeric repeats varied slightly, mostly by SNPs and single nucleotide indels. Overall, repetitive DNA made up ∼45% of the genome (Supplementary Table 2).

**Table 3.** Results of BUSCO genome assembly completeness analysis using the embryophyta_obd9 conserved ortholog data set.

No. genes	BUSCO category
1,318	Complete BUSCOs (91.5%)
1,215	Complete and single-copy BUSCOs (84.4%)
103	Complete and duplicated BUSCOs (7.2%)
33	Fragmented BUSCOs (2.3%)
89	Missing BUSCOs (6.2%)
1,440	Total BUSCO groups searched

**Fig. 2.**
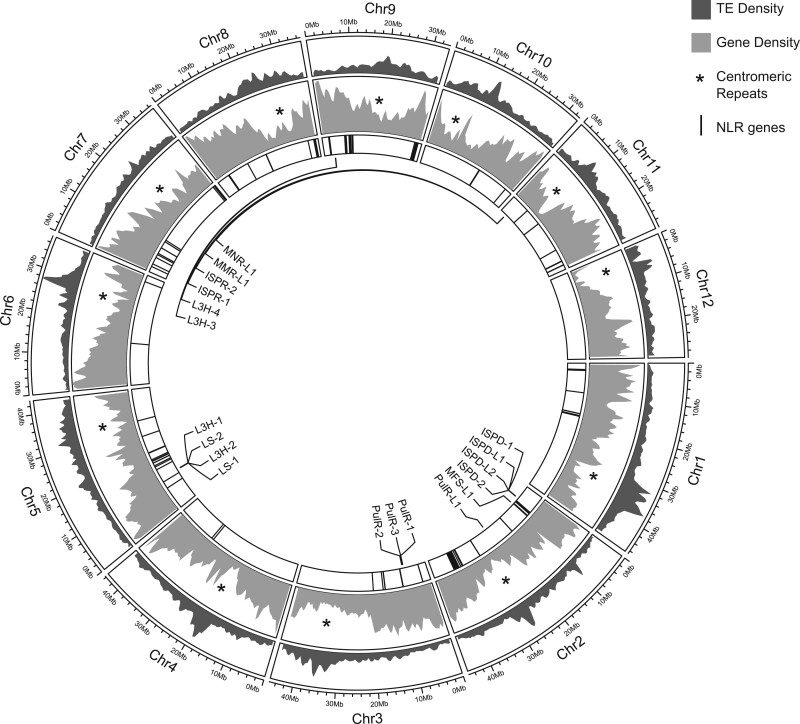
*Mentha longifolia* genome features. Chromosomal gene density profiles are shown in gray, with approximate centromere positions defined by tandem repeat analysis indicated with asterisks. Positions of NBS-LRR disease resistance gene homologs are indicated by vertical lines on the interior track. Monoterpene biosynthesis genes are labeled.

A total of 42,107 protein-coding genes were predicted in the v3.0 genome, an increase of 6,510 genes over the v1.0 draft assembly. A total of 37,190 (88.3%) v3.0 genes could be assigned to one or more Gene Ontology categories (complete list in Supplementary Table 3) A BUSCO analysis to assess genome completeness showed that, of the 1,440 genes in the embryophyta dataset, >91% were present, most of which were single-copy (Table 3). Gene density in putative centromeric regions was relatively lower than in other regions of chromosomes, as expected (Fig. 2).

A linkage map was constructed using 1,701 SNPs obtained from GBS data from an *M. longifolia* F2 population. GBS data were mapped to the v3.0 genome assembly to call SNPs, but genome scaffold and basepair positions of SNPs were anonymized prior to being used for linkage mapping. Only 0–3 SNPs mapped to any of the 314 extrachromosomal scaffolds in the genome assembly, and none could be incorporated into LGs. The linkage map was 1,184.5 cM in length, and consisted of 12 LGs ranging from 65 to 141.9 cM (Supplementary Fig. 2). Individual LGs contained 99–279 markers with an average spacing of 0.7 cM and a maximum spacing of 24.6 cM. The markers on each LG were cross-referenced back to their positions in the genome assembly to look for misassemblies. Median basepair distance between adjacent markers was 517 bp over the whole genome. Several dense clusters of SNPs occurred within 1 kb of each other on the genome assembly and mapped to the same centimorgan position. The precise order of markers clustered within regions smaller than 10 kb on chromosomal scaffolds could not be resolved on the LGs. However, no LGs on the final map contained markers from more than one chromosome, and no inversions or rearrangements were detected.

### Analysis of disease resistance genes

A total of 3,417 genes contained one or more domains conserved among plant disease resistance genes (Supplementary Table 4). There were 1,163 NBS domain-containing genes, of which 153 had both NBS and LRR domains (Table 4). This number is comparable with tallies of NLR genes reported for other dicot species (Table 4). All but one of the *M. longifolia* NLR genes were on chromosomal scaffolds (Fig. 2). The number of NLR genes varied widely among chromosomes (Fig. 2). Chromosome 12 was notable in that it did not have any gene annotations containing both NBS and LRR domains. However, there were 5 genes on chromosome 12 containing both NBS and transmembrane domains (Mlong585_39829, Mlong585_40449, Mlong585_40651, Mlong585_41847, and Mlong585_41850) and 88 genes with LRR domains in combination with kinase and/or transmembrane domains.

**Table 4.** Tally of nucleotide binding site (NBS) domain-containing proteins in the *M. longifolia* CMEN 585 v3.0 genome in comparison to *Fragaria vesca*, *Solanum lycopersicum*, and *Brassica oleracea*.

Protein domains present	*Mentha longifolia*	*Fragaria vesca* ^a^	*Solanum lycopersicum* ^b^	*Brassica oleracea* ^c^
TIR_NBS_LRR	0	23	26	121
Non_TIR_NBS_LRR	153	121	195	124
CC_NBS_LRR	54	60	195	47
NBS_LRR	99	61	NR^d^	77

a From Zhong et al. (2015).

b From Andolfo et al. (2014).

c From Bayer et al. (2019).

d Not reported.

CC, coiled coil; LRR, leucine-rich repeat; TIR, Toll-Interleukin-1-receptor-like.

Of the 153 NLR genes, 117 (76%) occurred in clusters, defined here as at least 3 NLR genes separated from each other by <1 Mb (Supplementary Table 5). Chromosomes 4, 6, 11, and 12 lacked R gene clusters. Chromosomes 8 and 10 each had a single cluster; chromosomes 1, 3, and 5 each had 2 clusters, and chromosomes 2, 7, and 9 each had 3 clusters, for a total of 19 clusters. Sixteen of the 19 clusters contained 3–7 genes and spanned regions of ∼40 kb to 1.53 Mb (Supplementary Table 5). There were 3 much larger clusters that stood out from the rest. Two of these were on chromosome 9, including the largest cluster by far, with 23 R genes spanning 2.75 Mb, and a 16-gene cluster spanning 1.14 Mb. Chromosome 9 had a total of 44 NLR genes, the most of any chromosome. Chromosome 2 was second in R gene tally, with 28 NLR genes. Chromosome 2 also had the third large R gene cluster, which had 17 NLR genes and, at 3.22 Mb, spanned the largest sequence space.

Thirty-four NLR genes occurred outside of clusters, either as singletons or gene pairs, and were located on all chromosomes except chromosome 9 and chromosome 12 (Supplementary Table 6). Chromosomes 2, 3, and 4 each had 1 R gene pair; chromosomes 7 and 8 each had 2 pairs, and chromosome 11 had 3 R gene pairs. Chromosomes 1,7, and 10 each had 1 R gene singlet; chromosomes 6, 8, and 11 each had 2 singlets, and chromosome 5 had a total of 5 singlets.

**Fig. 3.**
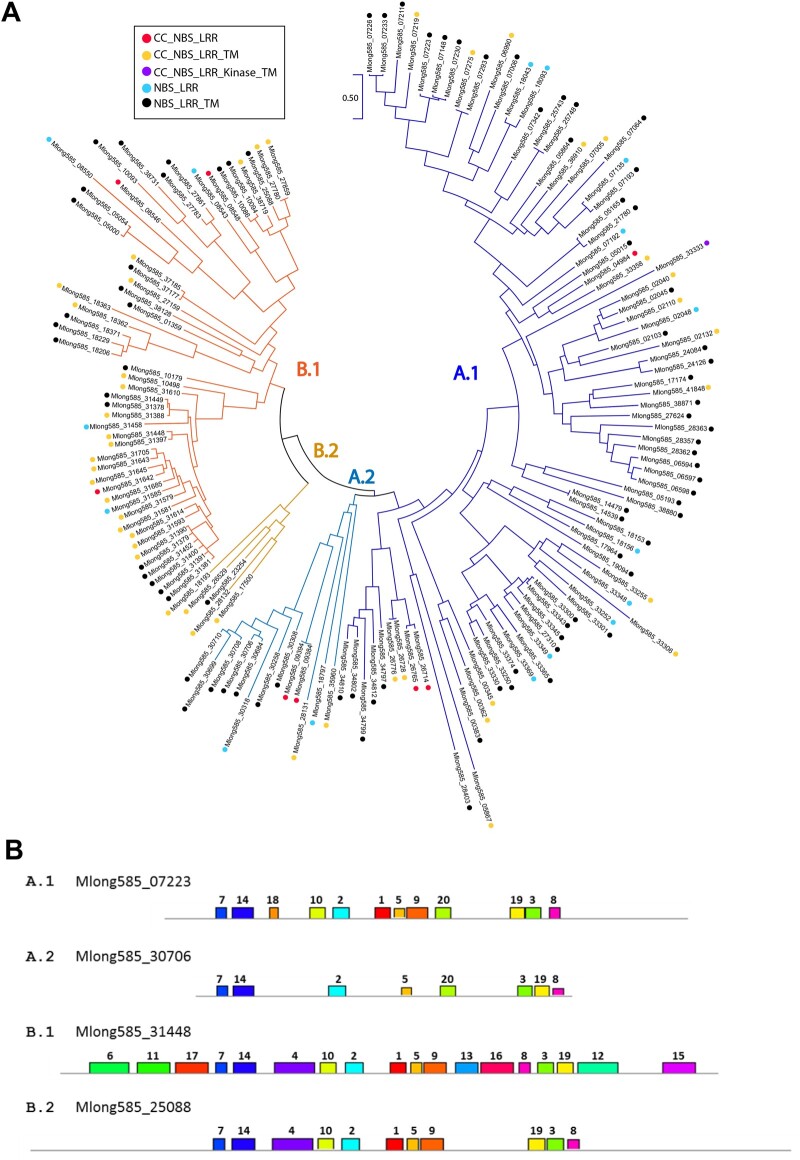
Maximum likelihood tree from predicted amino acid sequence alignment of *M. longifolia* disease resistance gene homolog nucleotide binding site domains.

**Fig. 4.**
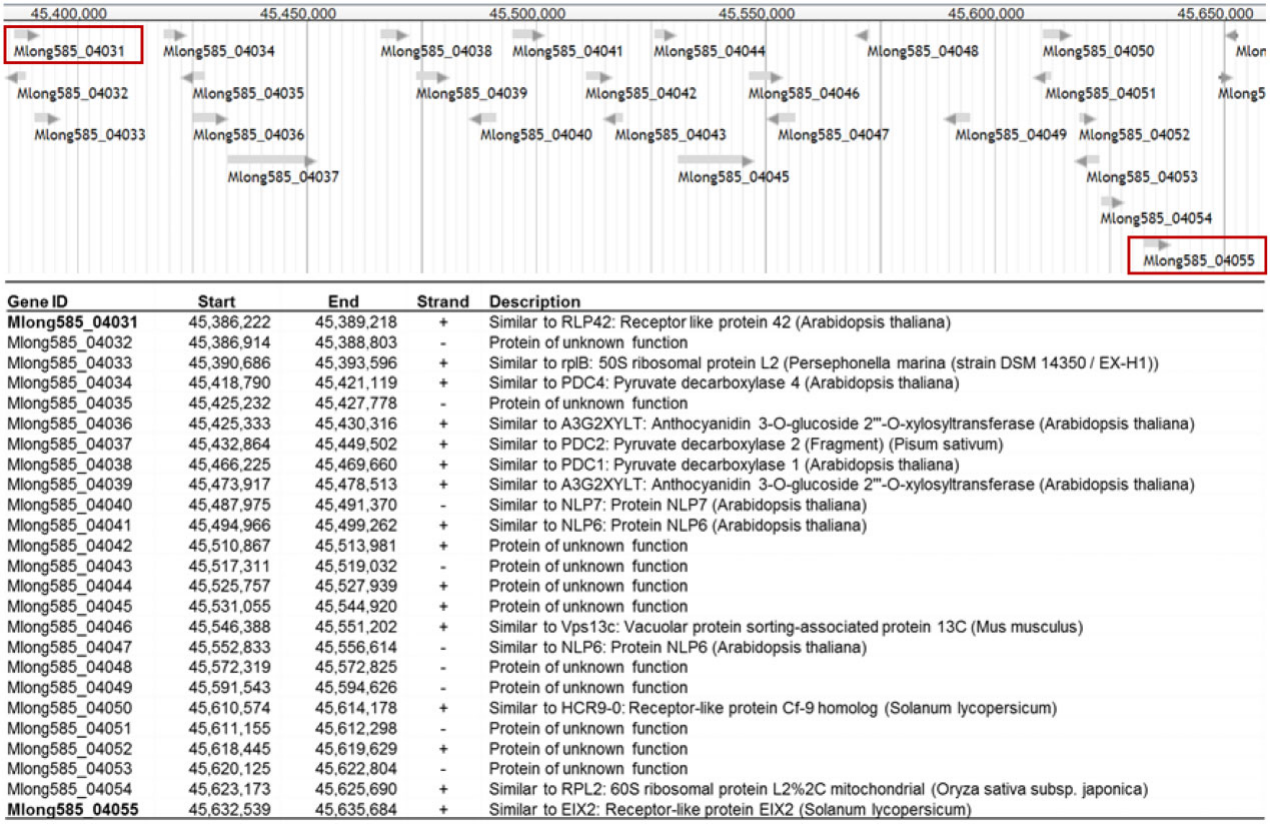
Amino acid sequence motifs enriched in 153 disease resistance gene homologs in the *M. longifolia* genome. a) Sequence logos of amino acid motifs enriched in the predicted nucleotide domains numbered i. Motifs Kinase-2, MHDV, P-loop, RNBS-A, RNBS-B, and RNBS-C have been previously described (Meyers *et al.* 1999, 2003). b) Representative nucleotide binding site (NBS) motif compositions of genes in 4 major grouping (Fig. 4).

Phylogenetic analysis of the predicted amino acid sequences of the NBS domains clustered the 153 NLR genes into 2 distinct groups (“A” and “B”), each containing 2 broadly defined subgroups (“A.1,” “A.2,” “B.1,” and “B.2”) (Fig. 3). The putative NBS amino acid sequences were then searched for characteristic NBS motifs. Representative motif composition of each subgroup is shown in Fig. 4 All 4 subgroups contained the P-loop and RNBS-A motifs (motifs 7 and 14). The “B” group contained a long (50 amino acid) motif that was comprised of adjacent Kinase-2 and RNBS-B motifs (motif 4). This motif was absent in the “A” group; however, subgroup A.1 contained a Kinase-2 domain that lacked an adjacent RNBS-B domain (motif 18). All 4 subgroups contained the “GLPL” domain (motif 2), and it was preceded by an RNBS-C domain in subgroups A.1, B.1, and B.2. The “A” groups and subgroup B.1 possessed different versions of the MHDV domain (motifs 20 and 13, respectively); this domain was absent in subgroup B.2. Subgroup A.1 and both “B” subgroups contained motifs 1, 5, and 9, while subgroup A.2 contained only motif 5. Three leucine-rich motifs, 19, 3, and 8, were present in all subgroups, but in different orders: subgroups A.1 and B.2 had these motifs in order 19, 3, 8; subgroup A.2. had the order 3, 19, 8 and subgroup B.1 had the order 8, 3, 19. Group B.1 was notable in that the predicted proteins possessed 5 conserved domains that were absent from all other groups. At the amino terminus, motif 11 was rich in the positively charged amino acids arginine and lysine. This motif was flanked on each side by motifs rich in negatively charged amino acids aspartate and glutamate (motifs 6 and 17). Motifs 12 (50 amino acids) and 15 (41 amino acids) were both present at the carboxy end of the NBS and may constitute transmembrane domains. Twenty-three of the 30 genes in this group belonged to the genome’s largest R gene cluster, on chromosome 9. Putative TIR domains were identified in 2 NBS-containing genes, Mlong585_05271 (506 bp) and Mlong585_04171 (749 bp). Neither gene contained LRR motifs, and both were short relatives to the NLR gene homologs. These may represent pseudogenes.

**Fig. 5.**
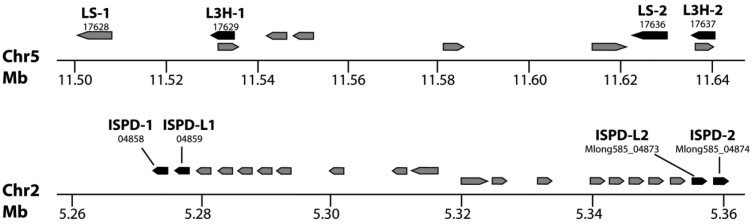
Chromosome 1 region containing putative homologs of tomato Ve1 with gene IDs, locations, and functional descriptions obtained from BLAST2GO.

To identify R genes with potential relevance to *Verticillium* wilt resistance, BLASTp searches against the *M. longifolia*_v3.0 genome were conducted using 6 genes involved in the tomato Ve1-mediated signaling pathway (Fradin *et al.* 2009) as queries. The closest mint homologs of these genes are shown in Table 5. Single homologs of *EDS1* and *NRC1* were found on chromosome 2. Single homologs of *SERK3*, *MEK2*, and *NDR1*were found on chromosomes 6, 7, and 10, respectively. One or more disease resistance-like motifs were found in all except the *EDS1* and *NDR1* homologs. Two homologs of *Ve1* were found on the (+) strand of *M. longifolia* chromosome 1, ∼246 kb apart (Fig. 5). The 2 genes, Mlong585_04031 and Mlong585_04055, were predicted to encode proteins of 1,016 amino acids and 976 amino acids, respectively. These genes shared 84% predicted amino acid sequence identity (89% positives) with each other, and 55–56% sequence identity (69–71% positives) with tomato Ve1 and Ve2 predicted proteins (Supplementary Fig. 3).

**Table 5.** Closest *M. longifolia* homologs of *S. lycopersicum* genes with reported roles in *Verticillium* wilt disease resistance signaling.

Canonical gene^a^	Mlong3.0 GeneID	Chromosome	Start	End	BLASTp % identities/ % positives
*Ve1*	Mlong585_04055	1	45,632,539	45,635,684	55%/69%
*Ve1*	Mlong585_04031	1	45,386,222	45,389,218	56%/69%
*EDS1*	Mlong585_04901	2	5,556,753	5,558,916	56%/75%
*NDR1*	Mlong585_35087	10	22,507,215	22,507,838	48%/64%
*NRC1*	Mlong585_06534	2	27,598,085	27,601,529	45%/63%
*SERK3*	Mlong585_21381	6	10,698,811	10,703,288	81%/86%
*MEK2*	Mlong585_27011	7	900,174	902,222	71%/77%

a From Fradin *et al.* (2009).

Percent identities refer to identical amino acid residues; percent positives refer to nonidentical substitutions that have a positive score in the underlying BLASTp scoring matrix.

### Chemical diversity of essential oils across *M. longifolia* accessions

Vining *et al.* (2005) performed a preliminary analysis of oils from *M. longifolia* accessions that tabulated major constituents. To better understand how the oil composition of the CMEN 585 accession correlates with chemical diversity across this species, we collected and analyzed oils from all *M. longifolia* accessions available through NCGR. Focusing on monoterpenoids, which constitute >95% of the oil (Supplementary Table 7), our data indicated the existence of 3 major and 2 minor chemotypes (Table 6). Four accessions accumulated >79% (+)-pulegone [CMEN 585, CMEN 700, CMEN 501, and CMEN 500; ordered by increasing (+)-pulegone content of up to 90%]. Seven accessions contained high quantities of the biosynthetically related constituents piperitone oxide and/or piperitenone oxide (CMEN 018, CMEN 017, CMEN 516, CMEN 635, CMEN 034, CMEN 020, and CMEN 592; ordered by increasing piperitenone content of up to 89%). (−)-Menthone and (−)-menthol were the signature constituents of a third chemotype (23% and 58%, respectively; CMEN 682). C6-oxygenated *p*-menthane monoterpenoids were dominant in 4 accessions, with 3 accumulating (−)-*trans*-carvone (up to 80%; CMEN 019, CMEN 584, and CMEN 707; fourth chemotype) and one containing high quantities of isodihydrocarveol and neoisodihydrocarveol (14% and 58%, respectively; CMEN 703; fifth chemotype) (Table 7). In summary, *M. longifolia* accessions produce remarkably diverse oils, resembling the chemotypic diversity observed with its sister species *M. suaveolens* (Vining *et al.* 2019). *Mentha* *longifolia* CMEN 585 (source of DNA for the chromosome-level assembly), CMEN 700, CMEN 501, CMEN 500, and CMEN 682 represent notable chemotypes because their oil composition more closely resembles that of *M.* × *piperita* (Lawrence 2007). We performed oil analyses for 13 *M. longifolia* accessions, which were generally consistent with data published previously (Vining *et al.* 2005). However, there were also minor discrepancies: in our analyses, accessions CMEN 020 and CMEN 592 both accumulated primarily piperitenone oxide and limonene, while they were reported previously to contain primarily pulegone and γ-muurolene, respectively. While we may not have a fully satisfying explanation for these differences, it should be noted that essential oil profiles are highly dependent on the conditions under which plants are grown. For the current study, plants were maintained in a greenhouse, but the growth and processing protocols were optimized to reflect the data that are typically obtained with field-grown plants (more details in Lange *et al.* 2011).

**Table 6.** Oil chemotypes of *M. longifolia* accessions.

Identifier in	Oil constituent [% of total monoterpenes]												
*Mentha* Collection	(+)- Pulegone	***cis*-Piperitone** **oxide**	*trans*- Piperitoneoxide	**Piperitenone** **oxide**	**(**−**)- Menthone**	**(**−**)- Menthol**	**(**−**)-*trans*- Carvone**	Isodihydro- carveol	Neoisodihydro- carveol	**(**−**)- Limonene**	1,8- Cineole	Piperitenone	Other
Pulegone accumulators													
CMEN585	79.34 ± 0.01	n.q.	0.09 ± 0.04	0.04 ± 0.05	0.06 ± 0.01	n.q.	n.q.	n.q.	n.q.	2.10 ± 0.15	3.67 ± 0.01	5.29 ± 0.03	9.40 ± 0.06
CMEN700	83.67 ± 0.02	n.q.	n.q.	n.q.	8.58 ± 0.01	n.q.	n.q.	n.q.	n.q.	0.47 ± 0.04	0.72 ± 0.14	0.49 ± 0.01	9.40 ± 0.25
CMEN501	85.89 ± 0.29	n.q.	n.q.	0.13 ± 0.02	3.08 ± 0.03	n.q.	n.q.	n.q.	n.q.	1.18 ± 0.22	0.69 ± 0.06	1.80 ± 0.05	7.22 ± 0.06
CMEN500	89.64 ± 0.41	n.q.	n.q.	0.05 ± 0.08	0.76 ± 0.19	n.q.	n.q.	n.q.	n.q.	0.73 ± 0.18	0.34 ± 0.12	0.60 ± 0.05	7.89 ± 0.08
Piperitone/piperitenone oxide accumulators													
CMEN018	n.q.	n.q.	81.89 ± 0.43	n.q.	n.q.	n.q.	n.q.	n.q.	n.q.	1.56 ± 0.25	2.47 ± 0.07	n.q.	14.07 ± 0.06
CMEN017	0.04 ± 0.03	19.59 ± 0.30	59.05 ± 0.37	4.08 ± 1.03	n.q.	n.q.	n.q.	n.q.	n.q.	2.45 ± 0.24	2.33 ± 0.19	0.13 ± 0.05	12.32 ± 0.18
CMEN516	n.q.	1.26 ± 0.16	39.21 ± 3.32	46.16 ± 4.36	n.q.	0.03 ± 0.04	n.q.	n.q.	n.q.	1.59 ± 0.54	0.51 ± 0.07	n.q.	11.24 ± 0.71
CMEN635	n.q.	9.78 ± 3.69	n.q.	78.65 ± 2.63	n.q.	n.q.	0.08 ± 0.02	n.q.	n.q.	2.02 ± 0.37	0.13 ± 0.01	0.67 ± 0.23	8.67 ± 0.58
CMEN034	0.01 ± 0.01	0.05 ± 0.03	0.56 ± 0.12	84.50 ± 0.98	n.q.	0.05 ± 0.01	0.04 ± 0.01	n.q.	n.q.	2.93 ± 0.63	0.10 ± 0.02	0.65 ± 0.07	11.13 ± 0.16
CMEN020	0.07 ± 0.12	n.q.	0.31 ± 0.27	88.14 ± 1.05	n.q.	0.14 ± 0.13	n.q.	n.q.	n.q.	2.83 ± 0.15	0.34 ± 0.06	1.43 ± 0.30	6.74 ± 0.17
CMEN592	n.q.	n.q.	0.54 ± 0.07	88.75 ± 0.01	n.q.	0.05 ± 0.04	n.q.	n.q.	n.q.	1.44 ± 0.36	0.15 ± 0.01	0.23 ± 0.08	8.84 ± 0.38
Menthone/menthol accumulators													
CMEN682	1.00 ± 0.04	0.09 ± 0.05	0.46 ± 0.01	n.q.	23.22 ± 0.01	59.32 ± 0.01	0.02 ± 0.01	n.q.	n.q.	0.90 ± 0.86	0.08 ± 0.03	0.16 ± 0.01	14.75 ± 0.10
Carvone accumulators													
CMEN019	0.05 ± 0.08	n.q.	n.q.	n.q.	0.17 ± 0.02	n.q.	66.54 ± 1.16	n.q.	n.q.	17.71 ± 0.85	3.37 ± 0.37	n.q.	11.81 ± 0.21
CMEN584	n.q.	n.q.	n.q.	n.q.	n.q.	n.q.	73. 71 ± 0.01	n.q.	n.q.	11.55 ± 0.27	5.74 ± 0.01	0.35 ± 0.01	8.63 ± 0.03
CMEN707	0.09 ± 0.04	n.q.	n.q.	n.q.	0.22 ± 0.01	n.q.	80. 21 ± 1.10	n.q.	n.q.	13.26 ± 0.15	0.54 ± 0.34	n.q.	5.68 ± 0.14
Isodihydrocarveol/neoisodihydrocarveol accumulators													
CMEN703	0.03 ± 0.04	0.05 ± 0.04	n.q.	0.03 ± 0.01	n.q.	0.21 ± 0.01	0.77 ± 0.35	14.44 ± 0.01	58.17 ± 0.01	11.19 ± 1.66	2.79 ± 0.03	n.q.	12.31 ± 0.35

Signature metabolites of each chemotype are indicated by gray background.

**Table 7.** Characteristics of gene families involved in essential oil biosynthesis in *M. longifolia*.

Gene annotation	*Mentha longifolia* chromosome	Location identifier	NCBI accession code	Number of introns	DNA strand	Expression in glandular trichomes [TPM]^a^	Homology to canonical genes (amino acid level)^b^
Terpene synthases (E.C. 4.2.3.- or EC 5.5.1.-)							
(−)-Limonene synthase							
LS-1	5	Mlong585_17628	MT664991	6	(−)	5,246	98% identity to Q40322
LS-2	5	Mlong585_17636	MT664992	6	(−)	As above	98% identity to Q40322
Oxidoreductases that incorporate 1 atom of oxygen (EC 1.14.13.- or EC 1.14.14.-)							
(−)-Limonene 3-hydroxylase							
L3H-1	5	Mlong585_17637	MT664993	1	(−)	2,281	99% identity to Q9XHE7
L3H-2	5	Mlong585_17629	MT664994	1	(−)	As above	99% identity to Q9XHE7
L3H-3	9	Mlong585_30867	MT664995	1	(+)	2,475	97% identity to Q9XHE7
L3H-4	9	Mlong585_30878	MT664996	1	(+)	As above	Pseudogene
(+)-Menthofuran synthase							
MFS-L1	2	Mlong585_05231	n.a.	2	(+)	1,180	90% identity to Q947B7
Oxidoreductases acting on CH-OH groups of donors (EC 1.1.1.-)							
(−)-Isopiperitenol/(−)-carveol dehydrogenase							
ISPD-1	2	Mlong585_04858	MT664997	0	(−)	137	98% identity to Q5C9I9
ISPD-2	2	Mlong585_04874	MT664998	0	(+)	As above	98% identity to Q5C9I9
ISPD-L1	2	Mlong585_04859	MT664999	0	(−)	127	80% identity to Q5C9I9
ISPD-L2	2	Mlong585_04873	MT665000	0	(+)	As above	80% identity to Q5C9I9
(−)-Menthone:(−)-menthol reductase							
MMR-L1	11	Mlong585_36607	n.a.	6	(−)	107	87% identity to Q9XHE7
(−)-Menthone:(+)-neomenthol reductase-like							
MNR-L1	11	Mlong585_36608	n.a.	0	(−)	n.a.	Pseudogene
Oxidoreductases acting on CH-CH groups of donors (EC 1.3.1.-)							
(−)-Isopiperitenone reductase							
ISPR-1	11	Mlong585_36604	MT665001	4	(−)	20	94% identity to Q6WAU1
ISPR-2	11	Mlong585_36605	MT665002	4	(+)	As above	99% identity to Q6WAU1
(+)-Pulegone reductase							
PulR-1	3	Mlong585_09229	MT665003	4	(−)	3,116	95% identity to Q6WAU0
PulR-2	3	Mlong585_09276	MT665004	4	(−)	As above	95% identity to Q6WAU0
PulR-3	3	Mlong585_09230	MT665005	0	(+)	n.a.	Pseudogene
PulR-L1	2	Mlong585_06346	MT665006	4	(−)	n.a.	83% identity to Q6WAU0

a Multimapped reads were counted once for each alignment and randomly assigned to the best alignment.

b Origin of reference sequences: spearmint, Q40322; peppermint, Q9XHE7, Q947B7, Q5C9I9, Q9XHE7, Q6WAU1, Q6WAU0.

n.a., not applicable; TPM, transcript per kilobase million.

### Functional annotation of genes involved in monoterpenoid essential oil biosynthesis

Building on the chemotyping data presented above, we investigated the *M. longifolia* CMEN 585 genome assembly for the presence of genes putatively involved in *p*-menthane monoterpenoid biosynthesis (organized below by class). The MAKER-P annotation pipeline identified 7 genes with sequence properties consistent with those of monoterpene synthases, which were grouped into 3 different clades by a sequence relatedness analysis (Supplementary Fig. 4). Two of these genes (identifiers Mlong585_17628 and Mlong585_17636); both located on chromosome 5, coded for enzymes with very high sequence similarity (98% identity at amino acid level) to the previously characterized LS of spearmint (Colby *et al.* 1993) (Table 7). The differences at the amino acid sequence level did not affect residues that were demonstrated to constitute the active site of LS (Srividya *et al.* 2015; Xu *et al*. 2018) and both LS-1 and LS-2 would therefore be expected to represent active isoforms. A recent publication reported on the functional characterization of LS-1 (Chen *et al*. 2021). A very high expression level of 5,246 TPM was determined for LS-1/LS-2 in an *M. longifolia* glandular trichome transcriptome data set acquired as part of the current study (due to extremely high sequence identity isoforms are only distinguishable in the genome assembly but not at the transcript level) (Table 7).

Genes encoding cytochrome P450-dependent monooxygenases (CYPs) with different regiospecificity had previously been characterized from peppermint [C3 hydroxylation by L3H (represented by the PM2 and PM17 cDNA clones)] and spearmint [C6 hydroxylation by L6H (represented by the SM12 cDNA clone)] (Lupien *et al.* 1999). Six *M. longifolia* genes were identified by the MAKER-P software as being related to these CYPs, and a sequence relatedness analysis separated them into 3 clades (Supplementary Fig. 4). Two of these genes [identifiers Mlong585_17629 (L3H-1) and Mlong585_17637 (L3H-2)], both located on chromosome 5, were close relatives of PM17 (99% identity at amino acid level). Two additional genes [identifiers Mlong585_30867 (L3H-3) and Mlong585_30878 (L3H-4); L3H-4 with premature stop codon], located on chromosome 9, shared very high homology with PM17 as well (97% identity at amino acid level). Interestingly, none of these genes were closely related to PM2 or SM12. Based on our *M. longifolia* glandular trichome transcriptome data, very high expression levels were determined for both L3H pairs (2281 TPM for L3H-1/L3H-2 and 2475 TPM for L3H-3/L3H-4; isoforms constituting each pair only distinguishable at the genome level) (Table 7). Interestingly, genes with high identity to L6H of *M. spicata* were absent in the *M. longifolia* genome, which is consistent with expectations based on oil constituents (C6-functionalized monoterpenoids below detection limit in *M. longifolia* CMEN 585 oil).

Genome annotation also identified 5 *M. longifolia* genes related to another CYP, the previously reported MFS of peppermint (Bertea *et al.* 2001), and these candidate genes separated into 3 clades in a sequence relatedness analysis (Supplementary Fig. 4). Only one of these genes (MFS-L1; identifier Mlong585_05231) had notable sequence homology (90% identity at the amino acid level) to the canonical MFS in a BLASTn search. This gene contained 2 introns and was located on chromosome 2 (Table 7). The corresponding transcript was expressed at high levels (1,180 TPM) in glandular trichomes. However, because of the comparatively high number of amino acid differences (affecting 49 amino acid residues) when compared with the canonical enzyme of peppermint (Supplementary Fig. 4) and the fact that (+)-menthofuran does not accumulate in *M. longifolia* CMEN 585, it will remain to be determined if MFS-L1 does indeed code for an enzyme with MFS activity.

The MAKER-P software identified 4 putative orthologs of the previously characterized ISPD gene from peppermint (Ringer *et al.* 2005), which separated into 2 clades in a sequence relatedness analysis (Supplementary Fig. 4). Very high sequence homology with the canonical ISPD was observed for 2 genes on chromosome 2 [identifiers Mlong585_04858 (ISPD-1) and Mlong585_04874 (ISPD-2); both have 98% identity to peppermint ISPD at the amino acid level] (Table 7). The sequences of ISPD-1 and ISPD-2 differ only by 2 nucleotides (A-G_287_ and T-C_307_ transitions) that result in nonsynonymous substitutions at the protein level (A to V and I to V, respectively). An expression level of 137 TPM was calculated for the corresponding transcript in glandular trichomes (isoforms only distinguishable at the genome level). The other 2 copies (identifiers Mlong585_04859 and Mlong585_04873), located in the same region on chromosome 2, had much lower homology to the canonical ISPD from peppermint (both 72% identity at amino acid level; designated as ISPD-L1 and ISPD-L2, respectively) (Table 7). The sequences of ISPD-L1 and ISPD-L2 differ by 2 nucleotides (C-T_76_ transition and G-C_121_ transversion) that result in nonsynonymous substitutions (L to F and E to Q, respectively). The expression levels of ISPD-L1/ISPD-L2 in glandular trichomes (127 TPM; isoforms only distinguishable at the genome level) was comparable to that of ISPD-1/ISPD-2. In vitro functional assays established that ISPD-L1 converted (−)-isopiperitenol to (−)-*trans*-isopiperitenone (Fig. 1), indicating that all 4 ISPD gene copies of *M. longifolia* are likely to be functional.

Based on the output of the MAKER-P gene annotation software, 17 putative orthologs of the ISPR gene of peppermint (Ringer *et al.* 2003), all on chromosome 11, were identified in the *M. longifolia* genome assembly (Supplementary Fig. 4), while reciprocal BLASTn searches of the canonical ISPR gene sequence against the *M. longifolia* CMEN585 genome sequence indicated that only 2 genes [identifiers Mlong585_36604 (ISPR-1) and Mlong585_36605 (ISPR-2)] shared very high homology with peppermint ISPR (94% and 99% identity at the amino acid level for ISPR-1 and ISPR-2, respectively) (Table 7). The expression level of ISPR-1/ISPR-2 (isoforms only distinguishable at the genome level) was low at 20 TPM. One gene with 87% identity (at the amino acid level) to the canonical MMR from peppermint (identifier Mlong585_36607; MMR-L1) was found to be located on chromosome 11 of the *M. longifolia* CMEN585 genome. The abundance of the corresponding transcript in glandular trichomes was modest (107 TPM) (Table 7). Both ISPR copies of *M. longifolia* were heterologously expressed in *E. coli*, and the corresponding recombinant proteins purified and assayed. Both were found to be active, converting (−)-isopiperitenone to (+)-isopulegone (Supplementary Fig. 4). A gene more closely related to mint MNR (identifier Mlong585_36608; MNR-L1) contained a premature stop codon and is thus considered a pseudogene. The highly homologous ISPR, MMR-L1 and MNR-L1 genes are situated in close vicinity in the genome sequence, thus indicating recent duplications.

Eight genes with homology to the previously reported PulR of peppermint (Ringer *et al.* 2003) were identified by the MAKER-P gene annotation package (Supplementary Fig. 4). Reciprocal BLASTn searches with the canonical PulR gene from peppermint indicated the presence of 2 genes with very high homology in the *M. longifolia* genome [identifiers Mlong_09229 (PulR-1) and Mlong_09276 (PulR-2); both with 95% identity to PulR at the amino acid level] (Table 7). The expression levels of these genes were low (20 TPM). A third putative ortholog (identifier Mlong_06346) had more sequence mismatches (83% identity to PulR at the amino acid level) and was therefore annotated as a PulR-like gene (PulR-L1). PulR-1 and PulR-2 were demonstrated in in vitro assays to convert (+)-pulegone into a mixture of (−)-menthone and (+)-isomenthone, whereas PulR-L1 was inactive against this substrate (Supplementary Fig. 5). A fourth gene copy [identifier Mlong_09230 on the (+)-strand (PulR-3; directly neighboring PulR-1) contained a premature stop codon and was thus annotated as a pseudogene (Table 7).

### Genomic organization of gene clusters related to monoterpenoid biosynthesis

The gene annotation for *M. longifolia* CMEN 585 revealed that oil biosynthetic genes can occur in discrete clusters. For example, chromosome 5 contains tandemly duplicated cassettes of the LS-1/L3H-1 and, at a distance of only about 84 kb, the LS-2/L3H-2 genes, both on the (−)-strand (Table 7 and Fig. 5). While the clustering of terpene synthases and CYPs, first observed in *A.* *thaliana* (Aubourg *et al.* 2002; Lange and Ghassemian 2003), was no surprise, we noted that the MAKER-P annotation pipeline also predicted the presence of open reading frames on the (+)-strand, transcribed in the opposite direction at the L3H-1 and L3H-2 loci, a genomic feature that has not been investigated previously in the context of terpenoid biosynthetic genes. Duplicated segments on opposite DNA strands were identified for larger genomic areas containing the ISPD-1/ISPD-L1 genes [chromosomal location 5,259,608–5,292,785 bp on the (−) strand] and ISPD-2/ISPD-L2 genes [chromosomal location 5,329,355–5,363,730 bp on the (+) strand], with a distance between segments of about 36 kb (Fig. 5).

## Discussion

Advances in molecular biology methods, combined with decreasing costs of DNA sequencing, have enabled chromosome-level assemblies for specialty crop plants with small research communities and limited resources. Indeed, as this trend continues, chromosome-level assemblies are becoming standard for diploid species, diploid phasing is increasing, and polyploid assemblies are rapidly improving. Here, we introduce the first chromosome-level genome assembly for an *M. longifolia* accession, a reference species for the diverse *Mentha* genus, as an important step toward learning about regulatory complexity of polygenic traits important to varietal improvement.

Linkage maps are useful for discovering errors in genome assemblies, particularly when assemblies are fragmented. The *M. longifolia* linkage map failed to incorporate any of the short extrachromosomal scaffolds into chromosomes because very few SNPs were called on the scaffolds, nor did it identify any regions where markers from different chromosomal scaffolds clustered together. The GBS method targets gene-rich regions, and the extrachromosomal scaffolds likely originated from more gene-sparse and/or repetitive regions. Overall, the linkage map presented herein is much improved compared with the earlier draft linkage map for this *M. longifolia* accession (Vining *et al.* 2017). The same GBS data used for the draft assembly were used to produce the new linkage map. The new linkage map incorporates 72 more markers than the draft linkage map. The previous map ordered 1,397 contigs into pseudochromosomes; the new map produced a single LG per chromosomal scaffold. For the previous linkage mapping effort, male-parent-derived and female-parent-derived markers were used to produce separate maps of 2,053.7 and 1,846 cM, respectively; these could not be merged because of a lack of collinearity. LGs in the new map incorporated markers from both parents, and the total map length was 661.5 cM shorter than the previous female parent map. The new map reflects the greater contiguity and accuracy of the Mlong v3.0 genome assembly, and its utility for study of clusters of disease resistance genes and monoterpene biosynthesis genes relevant to mint genetics and breeding.

### Diversity and genome organization of disease resistance genes in *M. longifolia*

NLR disease resistance gene homologs were unevenly distributed across the *M. longifolia* chromosomes, except for chromosome 12, which lacked these genes. Most of these genes were in clusters, although gene pairs and singletons were also present. The size and distribution of clusters is similar to what has been observed in diverse plant species (Dolatabadian *et al.* 2020; Mizuno *et al.* 2020), supporting the predominant model of R gene evolution driven by tandem duplication as well as larger-scale segmental duplications followed by localized rearrangements (Meyers *et al.* 2003; Leister 2004). Genome-wide studies in rice and *Arabidopsis* have shown that NLR genes in clusters are more polymorphic than singletons because of localized rearrangements (Yang *et al.* 2006; Guo *et al.* 2011). While the canonical “gene for gene” disease resistance model considers R gene function on the basis of single R genes, the emerging picture is one of greater complexity, such that in some cases the disease resistance response may occur at the pair or cluster level. For example, a *Eucalyptus* study revealed expression hotspots at R gene clusters, including expression of pseudogenes, indicating a role of physical proximity (Christie *et al.* 2015). In *Arabidopsis*, function of the temperature-sensitive defense response gene CHS1 requires the adjacent SOC3 gene (Zhang *et al.* 2017).

TNL genes were absent in the *M. longifolia* genome. There were 2 genes with putative TIR domains; however, while these 2 genes had both TIR and NBS domains, they lacked LRR or kinase domains. The absence of LRR or kinase domains, combined with the relatively short lengths of the 2 genes, make it less likely that these are functional disease resistance genes. The TNL, CNL, and RNL subfamilies represent anciently diverged lineages with distinct evolutionary histories (Cannon *et al.* 2002; Shao *et al.* 2016; Monteiro and Nishimura 2018). TNLs appear to be absent in monocots, and their presence or absence in particular dicot genera does not show a clear evolutionary path (Monteiro and Nishimura 2018). To date, *M. longifolia* has been the subject of the only study of NLR genes in *Mentha* or any Lamiaceae genus (Vining *et al.* 2007), and 3 sequences from that study were included in the only comprehensive phylogenetic analysis of NLR genes studies to date that has included Lamiaceae (McHale *et al.* 2006). The work presented here represents the first representation of the full complement of disease resistance genes and their organization in the Lamiaceae, providing the foundation for comparative R gene studies within this family.

Comparisons of R genes among *Mentha* species may provide insights into the origin and genome composition of “Black Mitcham” peppermint. This hexaploid mint’s ancestry includes genome contributions from 3 distinct *Mentha* species: *M. longifolia*, *M. suaveolens*, and *M. aquatica.* “Black Mitcham” is sexually sterile, meaning there has been no opportunity for chromosomal translocations or other rearrangements. Species-specific NLR gene duplication events have been observed among species within *Arabidopsis* (Mondragón-Palomino *et al.* 2017) *Solanum* (Seong *et al.* 2020), and *Fragaria* (Zhong *et al.* 2018). If such species-specific duplications exist among the 3 ancestral *Mentha* species, they could potentially be traced to the subgenomes of “Black Mitcham.”

*Verticillium* wilt resistance mediated by Ve1 and downstream signaling proteins is best studied in the Solanaceae (Fradin *et al.* 2009; Zhang *et al.* 2013; Fradin *et al.* 2014; Liebrand *et al.* 2014). In tomato, the *Ve* locus was shown to contain 2 closely linked, inverted genes, *Ve1* and *Ve2* (Kawchuk *et al.* 2001). *Ve1*, but not *Ve2*, was shown to confer *Verticillium* wilt resistance in tomato and *Arabidopsis* (Fradin *et al.* 2011). Ve1 homologs have been identified in several other plant families, indicating the early evolution of this disease resistance pathway (Song *et al.* 2017). Mint Ve1 homologs were first identified in *Mentha* USDA accessions in 2009 (Vining and Davis 2009). In the *M. longifolia* v3 reference genome, 2 mint Ve homologs are on chromosome 1, and, like the tomato Ve genes, they share ∼84% sequence identity. Unlike tomato, the 2 mint Ve genes are >240 kb apart on the same strand. More than 20 genes were annotated between them, among them a homolog of the tomato Cf-9 *Cladosporum fulvum* fungal disease resistance gene. Annotation of mint homologs of genes acting downstream of Ve indicates the possibility that a functional Ve signaling pathway exists in mint. Given the crosstalk among plant disease resistance signaling pathways (Fradin *et al.* 2009), the specificity of such genes’ functions remains to be seen. Determining whether alleles of these genes are expressed in response to *Verticillium* challenge will be an important step in providing functional annotations, and in developing a mechanistic model for mint *Verticillium* wilt resistance. Altogether, this study provides the foundation for ongoing work in mint breeding and comparative disease resistance genomics within *Mentha* and the broader Lamiaceae.

Breeding for *Verticillium* wilt disease resistance is an immediate goal of the US mint industry. Durable disease resistance to *Verticillium* wilt and other diseases using multiple genetic sources is a long-term goal. With this goal comes a need to carefully curate the complement of disease resistance genes in *Mentha* species germplasm. The catalog of NLR disease resistance gene homologs in CMEN 585 adds to the growing body of annotated plant “resistomes,” enabling taxonomic comparisons. These mint annotations will aid studies of gene expression in response to *V. dahliae* and other pathogen challenges.

### Genomic organization of oil biosynthetic genes hints at possibility of epigenetic regulation

An analysis of genomic organization in *M. longifolia* indicated that some oil biosynthetic genes occur in discrete clusters (e.g. tandemly duplicated LS/L3H genes and a duplicated segment containing ISPD copies). The presence of recently duplicated, paralogous genes indicate the possibility of epigenetic regulation: microRNAs have been implicated as one of the primary mechanisms of subfunctionalization or neofunctionalization of duplicated genes (Maher *et al.* 2006; Nozawa *et al.* 2012; Wang and Adams 2015). The clustered LS/L3H segments also contained overlapping open reading frames on the opposite strand, which could lead to the formation of natural antisense transcripts that can exert regulatory functions (Ariel *et al.* 2015). The regulatory cascade involving microRNAs can also lead to changes in DNA methylation, which can then affect gene expression. For example, we previously demonstrated that spearmint had very high expression levels of several genes known to be involved in the biosynthesis of C3-oxygenated monoterpenes, although it does not accumulate the corresponding products in the oil (spearmint produces C6-oxygenated monoterpenes) (Ahkami *et al.* 2015). There was one notable exception: the ISPR gene of the C3 oxygenation pathway was not expressed in spearmint. To shed light on the potential cause of ISPR suppression in spearmint, we obtained sequence upstream of ISPR from genomic DNA of several mint cultivars. Interestingly, all mint genomes had an arrangement in which an ISPR-like gene was positioned in reverse orientation at the 5′-end of the promoter. It had been shown previously that such an inverted repeat can be linked to gene silencing (Guo *et al.* 2016). Based on our hypothesis that the ISPR gene might be repressed in spearmint by epigenetic mechanisms, we performed bisulfite sequencing (Ahkami *et al.* 2015). When whole leaves were used for this analysis, a methylation rate of ∼20% was detected for the ISPR coding sequence for all cultivars. In contrast, the total cytosine methylation rate of the ISPR gene in genomic DNA isolated from glandular trichomes was significantly higher in spearmint compared with watermint and peppermint, indicating cell type-specific differences across cultivars (Ahkami *et al.* 2015). With reference genomes and transcriptomes now being available for *M. longifolia*, assessing epigenetic control of oil biosynthesis will be an exciting future prospect for furthering our understanding of the regulation of essential oil biosynthesis, with broader implications beyond mint.

### Teasing out the functions of genes involved in *p*-menthane monoterpenoid biosynthesis, with an emphasis on a family of multifunctional reductases

We performed oil analyses for 13 *M. longifolia* accessions, which were generally consistent with data published previously (Vining *et al.* 2005). However, there were also minor discrepancies: in our analyses, accessions CMEN 020 and CMEN 592 both accumulated primarily piperitenone oxide and limonene, while they were reported previously to contain primarily pulegone and γ-muurolene, respectively. We do not have a satisfying explanation for these differences but are highly confident in the data presented here, which rely on annotation based upon rigorous analyses with a method that provides an exquisite separation of mint oil constituents and a comprehensive in-house library of authentic standards. *Mentha* *longifolia* CMEN 585 accumulates (+)-pulegone as the dominant monoterpenoid in the oil (79%). Other constituents of note are piperitenone (5%) and 1,8-cineole (4%). Both (+)-pulegone and piperitenone are C3-functionalized monoterpenoids derived from a common, branched pathway, which will be discussed in depth below. The formation of 1,8-cineole from geranyl diphosphate is catalyzed by a dedicated monoterpene synthase in other plants (Wise *et al.* 1998; Chen *et al.* 2004; Shimada *et al.* 2005; Roeder *et al.* 2007; Fähnrich *et al.* 2011; Demissie *et al.* 2012). A recent survey of the *M. longifolia* genome unearthed 63 candidate genes for terpene synthases but a prediction which of these might code for a 1,8-cineole synthase was not provided (Chen *et al.* 2021).

Genes with very high identity to those known to be required for the biosynthesis of (−)-menthone in *M. piperita* (Fig. 1), in particular those putatively coding for LS, L3H, ISPD, ISPR, and PulR, were present in the *M. longifolia* genome (Table 7). *Mentha* *longifolia* genes encoding isoforms of ISPD, ISPR, and PulR were functionally characterized in follow-up experimental research presented here. The fact that products downstream of (+)-pulegone, namely (−)-menthone and (−)-menthol, predominate in peppermint is likely due to the lack of a (−)-*trans*-isopiperitenone isomerase (ISPI) activity, which prevents the formation of products of the piperitenone branch. Future research will have to test this hypothesis. Our results further underscore the complexity of monoterpenoid biosynthesis in mint, which has been demonstrated to involve multiple levels of control, including differential gene expression, enzyme abundance, feedback control, and epigenetic mechanisms (McConkey *et al.* 2000; Rios-Estepa *et al.* 2008, 2010; Ahkami *et al.* 2015).

## Data availability

The genome data generated in this study have been submitted to the NCBI Eukaryotic Genomes database under accession number PRJNA310613. A sorted listing of accession numbers for genes involved in *p*-menthane monoterpene biosynthesis is provided in Table 7.

Supplemental material is available at *G3* online.

## Funding

This work was funded by the Mint Industry Research Council (grants to KJV and BML) and the Oregon Mint Commission (grant to KJV). Work to identify monoterpene synthase genes was supported the Division of Chemical Sciences, Geosciences, and Biosciences, Office of Basic Energy Sciences, and US Department of Energy (grant no. DE-SC0001553 to BML).

## Conflicts of interest

None declared.

## Supplementary Material

jkac112_Legends_for_Supporting_Information

jkac112_Figure_S1

jkac112_Figure_S2

jkac112_Figure_S3

jkac112_Figure_S4

jkac112_Figure_S5

jkac112_Figure_S6

jkac112_Table_S1

jkac112_Table_S2

jkac112_Table_S3

jkac112_Table_S4

jkac112_Table_S5

jkac112_Table_S6

jkac112_Table_S7
