# Supplementary material for: Chromosome-level genome assembly of Mentha longifolia L. reveals gene organization underlying disease resistance and essential oil traits
Source: G3 (Bethesda). 2022 May 12;12(8):jkac112. doi: 10.1093/g3journal/jkac112 (PMC9339296; doi:10.1093/g3journal/jkac112)
Supplement: jkac112_Legends_for_Supporting_Information [file jkac112_legends_for_supporting_information.docx]

**Legends for Supporting Information**

**Table S1.** Genome alignment statistics of different versions of the *M. longifolia* genome assembly.

**Table S2.** Repetitive sequence complement of the *M. longifolia* v3.0 genome.

**Table S3.** Complete list of Gene Ontology categories assigned to annotated protein-coding genes in the *M. longifolia* v3.0 genome.

**Table S4.** Tally of genes containing one or more protein domains common to plant disease resistance genes in the *M. longifolia* v3.0 genome.

**Table S5.** Positions of NBS-LRR gene clusters on *M. longifolia* chromosomes.

**Table S6.** Positions of NBS-LRR gene pairs and singletons on *M. longifolia* chromosomes.

**Table S7.** Relative quantities of individual monoterpenes in USDA *M. longifolia* accessions.

**Figure S1.** Representative centromeric repeat sequences from each of the 12 M. longifolia chromosomes.

**Figure S2.**  Genetic linkage map showing distribution of SNP markers on assembled Mlong v3.0 chromosomes. The x-axis shows numbering of linkage groups relative to chromosomes.

**Figure S3.** Alignment of predicted amino acid sequences from Ve genes of mint and tomato. A. Alignment of two *M. longifolia* Ve homologs. B, C. Alignment of each predicted mint Ve protein with tomato Ve1 and Ve2.

**Figure S4.** Neighbor-joining tree showing sequence similarity among *M. longifolia* monoterpene biosynthesis gene orthologs.

**Figure S5.** GC-FID chromatograms of enzyme assay substrates and products obtained as part of efforts to characterize enzymes involved in monoterpenoid biosynthesis in *M. longifolia*.
