# Supplementary figures and images for: Chromosome-level genome assembly of Mentha longifolia L. reveals gene organization underlying disease resistance and essential oil traits
Source: G3 (Bethesda). 2022 May 12;12(8):jkac112. doi: 10.1093/g3journal/jkac112 (PMC9339296; doi:10.1093/g3journal/jkac112)

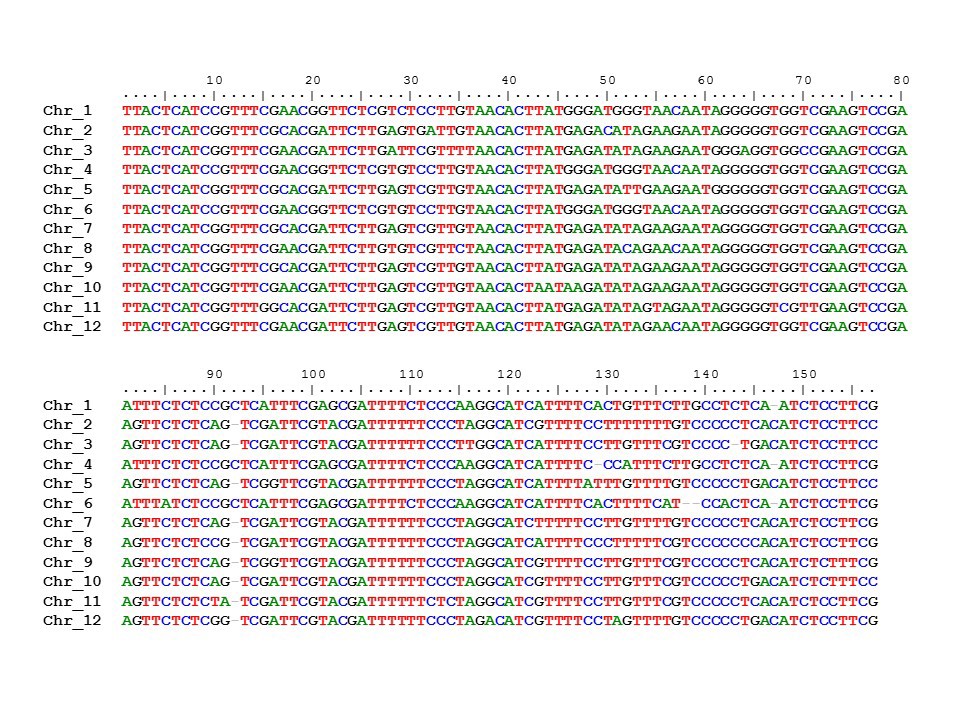

Supplement: jkac112_Figure_S1 [file jkac112_figure_s1.jpeg]

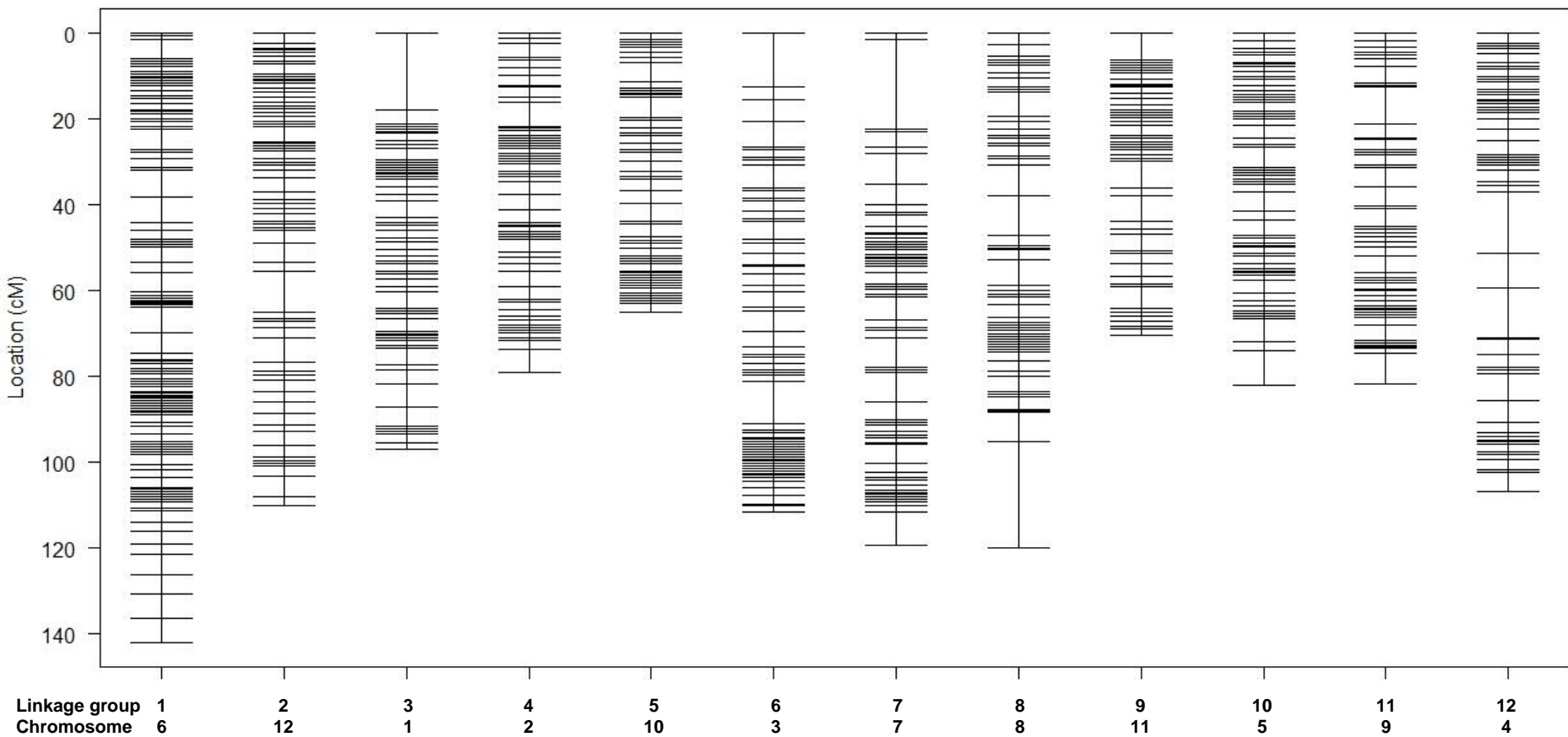

Supplement: jkac112_Figure_S2 [file jkac112_figure_s2.pdf]

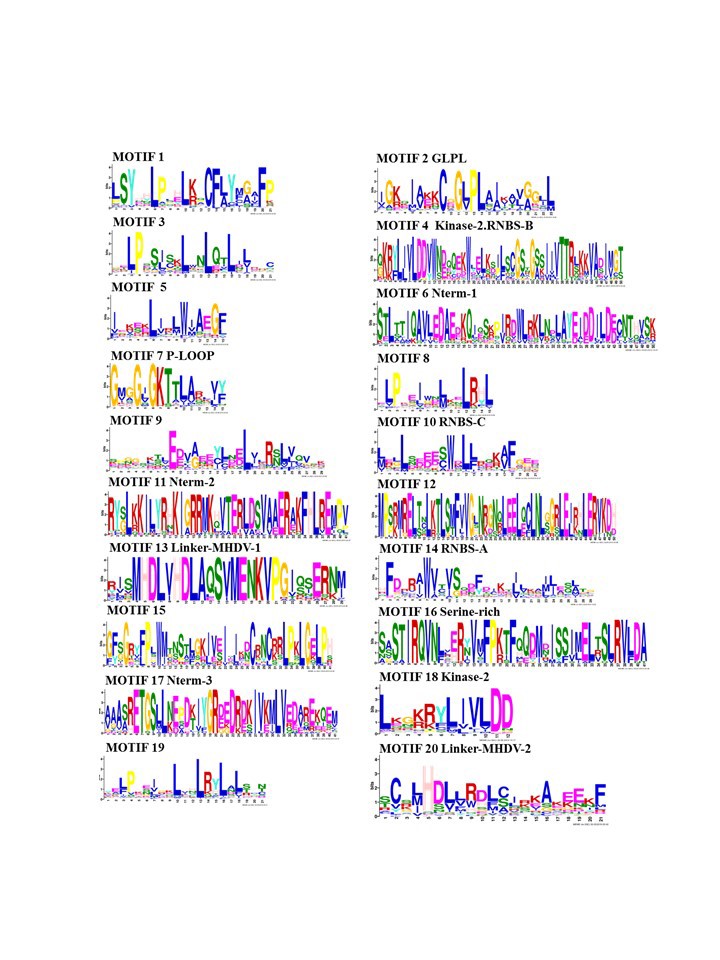

Supplement: jkac112_Figure_S3 [file jkac112_figure_s3.jpeg]

LS

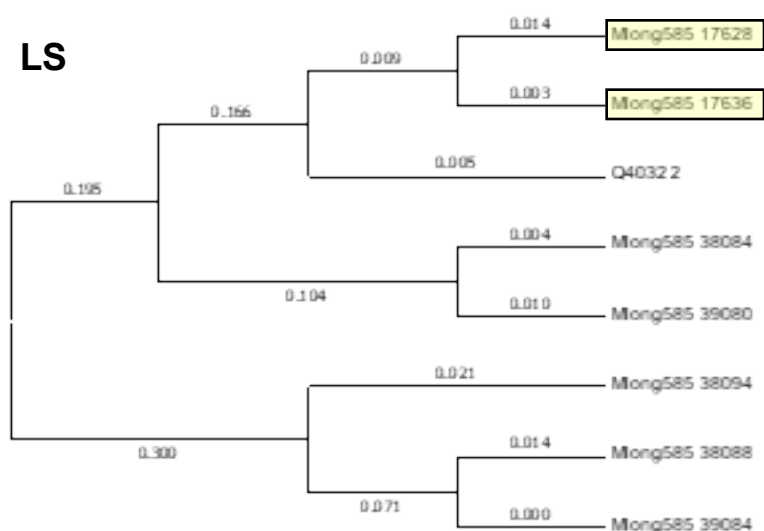

L3-H

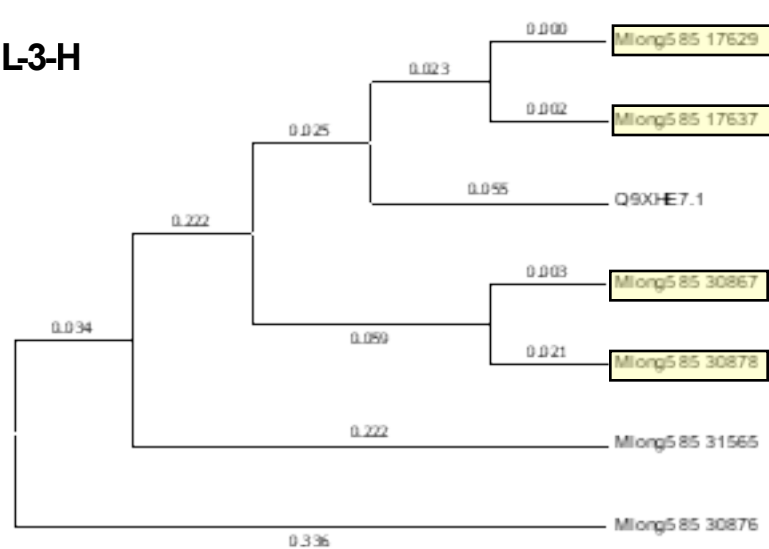

MFS

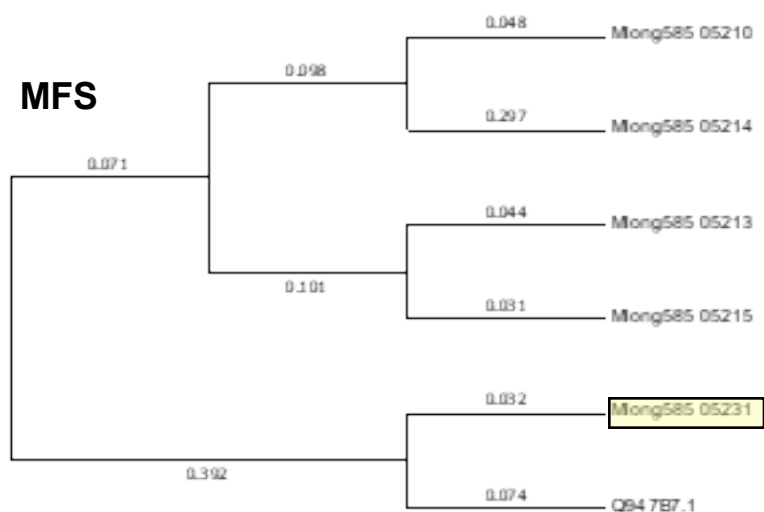

ISPD

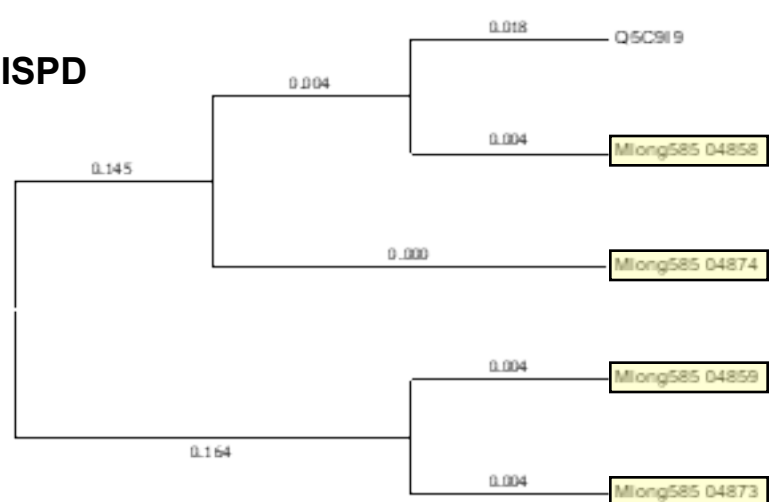

ISPR

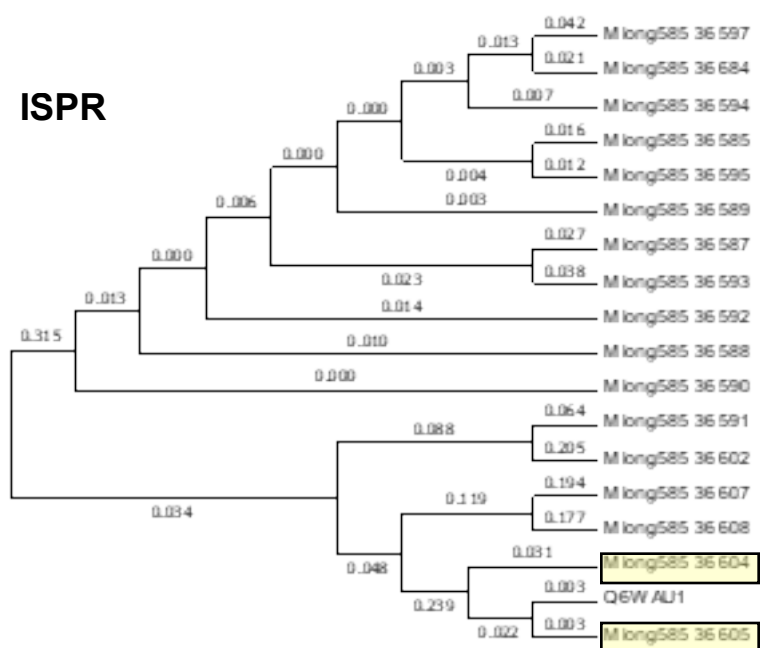

PR

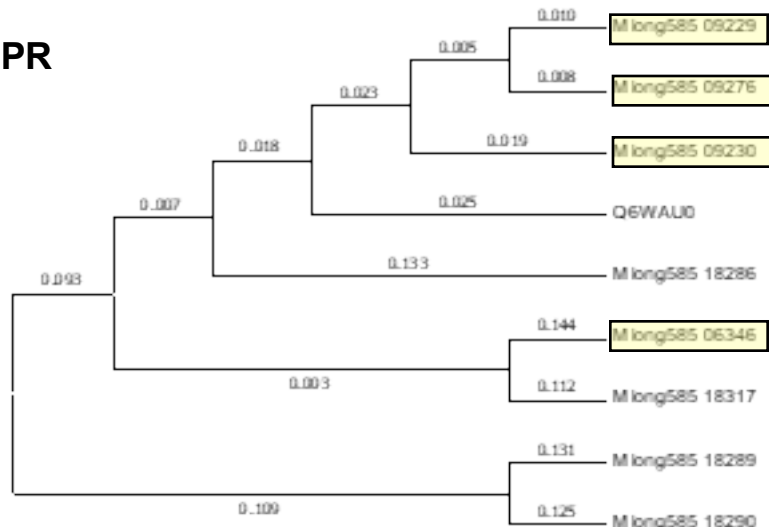

MMR

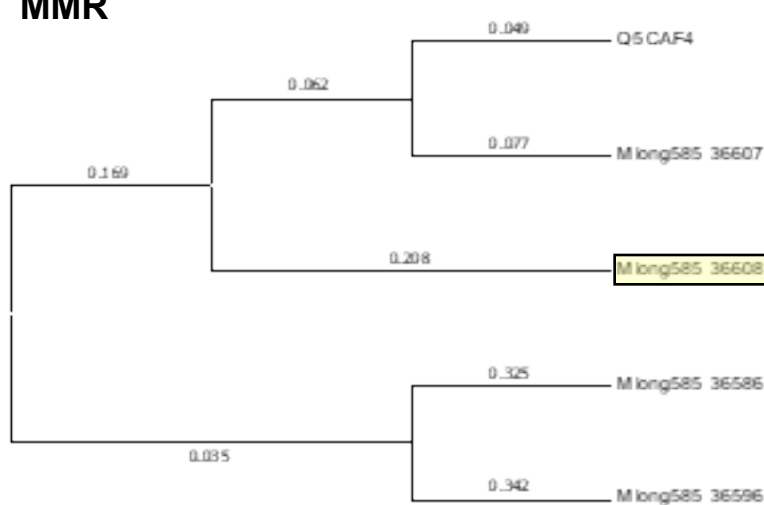

MNR

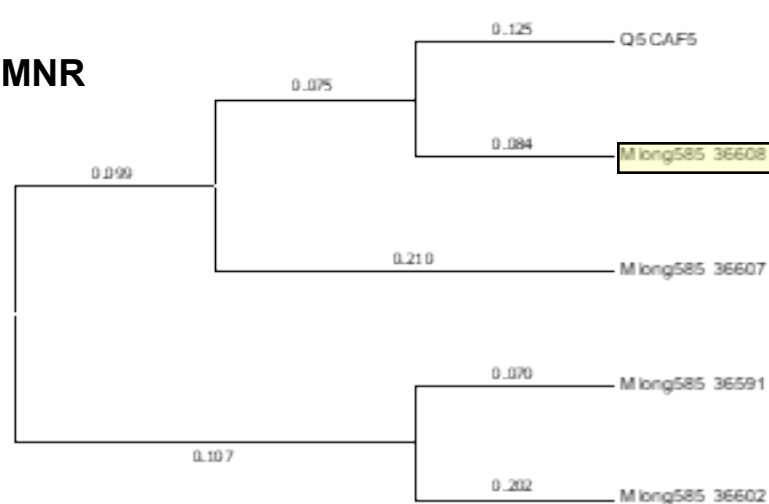

Supplement: jkac112_Figure_S5 [file jkac112_figure_s5.pdf]
