# Supplementary material for: Chromosome-level genome assembly of Mentha longifolia L. reveals gene organization underlying disease resistance and essential oil traits
Source: G3 (Bethesda). 2022 May 12;12(8):jkac112. doi: 10.1093/g3journal/jkac112 (PMC9339296; doi:10.1093/g3journal/jkac112)
Supplement: jkac112_Figure_S4 [file jkac112_figure_s4.pdf]

A.

```
                          10        20        30        40        50        60        70
                  ....|....|....|....|....|....|....|....|....|....|....|....|....|....|
Mlong585_04031    MRIRGLPWFLFISLIQILFLAGVYTQCLDDQRFILLQLKSALAFDLTSSTKL-----------WNGVDCD
Mlong585_04055    ...QLFSRL.........I.VL.SS...E...SS.....NT.E.NSN..S..VKWNQSDECCR.E.....

                          80        90       100       110       120       130       140
                  ....|....|....|....|....|....|....|....|....|....|....|....|....|....|
Mlong585_04031    GAGHVISLNLESETISGGIENSSTLFRLQYLEELNLAFNFFNSDQIPKGFQNLTSLSYLNLSNAGFAEQV
Mlong585_04055    .E.R..G....N...T...N...P..E....KR.........GA.....I....N.AH........GG..

                         150       160       170       180       190       200       210
                  ....|....|....|....|....|....|....|....|....|....|....|....|....|....|
Mlong585_04031    PIEVSMMSSLIVLDLSTLFGGAQPLKLEKPNLQQLAQNLTGLRELYLDNVNISSQRSEWGPALSSSLPNL
Mlong585_04055    .V.I.RL...LI.........P...R..N....R.V...PR.......Y..........CQ.........

                         220       230       240       250       260       270       280
                  ....|....|....|....|....|....|....|....|....|....|....|....|....|....|
Mlong585_04031    TTLSLRSCGLSGPLDSSLAGFHSLSVLRLDDNNLSATVPEFMSGFSNLTALNLGNCFLQGTFPDKIFQLH
Mlong585_04055    ..................GDLR...I....R....TMI.V..AD.....T.T..S...L.........VK

                         290       300       310       320       330       340       350
                  ....|....|....|....|....|....|....|....|....|....|....|....|....|....|
Mlong585_04031    TLQVLDLSNNKNLNGAIPEFPVSSSLRTMILSYTNFSGWLPNSIGNLRMLSKIDFSNCNFTGSIPASVSN
Mlong585_04055    ...N.E....EQ...T...L.........V........S..H.........R..L............I..

                         360       370       380       390       400       410       420
                  ....|....|....|....|....|....|....|....|....|....|....|....|....|....|
Mlong585_04031    LTELVHLDFSLNDFMGSIPPFSMSKKLAYLDLSRNSITGSLSSKHFEGLSNLVYINLGVNSLNGTIPPSL
Mlong585_04055    ............S.....H........V........L......Q..........................

                         430       440       450       460       470       480       490
                  ....|....|....|....|....|....|....|....|....|....|....|....|....|....|
Mlong585_04031    FDLPSLQRLHLFNNKFSGQITEFPIPNPSSLDTVDLSNNRLEGSIPESFFKLEGLSILSLSSNLFNGSLK
Mlong585_04055    .G.....S.Q.S..R.....G........T...L...S...D.............NV........H.T..

                         500       510       520       530       540       550       560
                  ....|....|....|....|....|....|....|....|....|....|....|....|....|....|
Mlong585_04031    LEKVQRFPNLTRLELGYNRLSVDTSNMSSFQYPQLSRLNLASCNLLDFPNLRNQSRLLFLDLSHNSIKGE
Mlong585_04055    .....S............K...................................................

                         570       580       590       600       610       620       630
                  ....|....|....|....|....|....|....|....|....|....|....|....|....|....|
Mlong585_04031    LPSWIWNIGNGSLNHLNVSRNYLVDLQKPFTIPPFLGVLDLNTNSLKGEFPLPPPSATYVDYSNNSFQQG
Mlong585_04055    .................L.......................................I...........A

                         640       650       660       670       680       690       700
                  ....|....|....|....|....|....|....|....|....|....|....|....|....|....|
Mlong585_04031    IPVNVANNSLTGTIPESLCDATYLQVLDLSDNNLSGTIPDCLVKGFTLAVLNLRRNNIEGNIPDVFSSTC
Mlong585_04055    ......................................................................

                         710       720       730       740       750       760       770
                  ....|....|....|....|....|....|....|....|....|....|....|....|....|....|
Mlong585_04031    GLKTLDLSQNNIGGKIPLSMANCTSLEVLNIGNNKIEDEFPCRLKSSSSLRVLVLRSNKLYGDISCTEIN
Mlong585_04055    ...........................I..V.......V....................F..N.......

                         780       790       800       810       820       830       840
                  ....|....|....|....|....|....|....|....|....|....|....|....|....|....|
Mlong585_04031    GSWPNLQIIDIAFNSFGGEINPRCIASWKGMMLGNGTQLRGEHIRFEFLTKFYYQDAVTVTIKGLELELV
Mlong585_04055    .T................K.......T..............D.......K------LM............

                         850       860       870       880       890       900       910
                  ....|....|....|....|....|....|....|....|....|....|....|....|....|....|
Mlong585_04031    KILTLFTAIDLSCNNFSGNIPQTIGDLSSLYVLNLSHNAFSGAIPSSIGNLKQLGSLDLSVNQLTGMVPQ
Mlong585_04055    ....V.....F...K......P.V.......I......S......R........................

                         920       930       940       950       960       970       980
                  ....|....|....|....|....|....|....|....|....|....|....|....|....|....|
Mlong585_04031    ELTSLNFLSFLNLSYNMLVGKIPDGNQLQTFSALSYTGNLGLCGFP--LMNTICYHNSPVGNSP------
Mlong585_04055    ....................................I..V.YPAK.TKRW.S.DFNWQF.FTGLGFGVGA

                         990      1000      1010      1020
                  ....|....|....|....|....|....|....|....|..
Mlong585_04031    --------------ASLNKRVYDWQLIIT-------------
Mlong585_04055    SLILAPLAFCKEWREKCDEQTDQFMKM.YPKYGFSYVRHDSK
```

B.

```
                            10        20        30        40        50        60        70
                    ....|....|....|....|....|....|....|....|....|....|....|....|....|....|
Mlong585_04055      ---MRIQLFSRLLFIS-LIQILI---LVLVSSQCLEDQRSSLLQLKNTLEFNSNSSSKLVKWN-QSDECC
Ve1[AAK58682.1]     MKM.ATLY.PMV.L.P-SF...SGYHIF......D..K.L...F.GS.QYD.TL.K..A...DMTS...

                            80        90       100       110       120       130       140
                    ....|....|....|....|....|....|....|....|....|....|....|....|....|....|
Mlong585_04055      RWEGVDCDGEGRVIGLNLENETITGGINNSSPLFELQYLKRLNLAFNFFNGAQIPKGIQNLTNLAHLNLS
Ve1[AAK58682.1]     N.N..T.NLF.H..A.E.DD...SS..E...A..S....ES....D.M..VG-..V..D.....KY....

                           150       160       170       180       190       200       210
                    ....|....|....|....|....|....|....|....|....|....|....|....|....|....|
Mlong585_04055      NAGFGGQVPVEISRLSSLLILDLSTLFGG-PQPLRLENPNLQRLVQNLPRLRELYLDYVNISSQRSEWCQ
Ve1[AAK58682.1]     ....V..I.ITL...TR.VT.....ILPFFD...K......SHFIE.STE.......G.DL....T....

                           220       230       240       250       260       270       280
                    ....|....|....|....|....|....|....|....|....|....|....|....|....|....|
Mlong585_04055      ALSSSLPNLTTLSLRSCGLSGPLDSSLGDLRSLSILRLDRNNLSTMIPVFMADFSNLTTLTLGSCFLLGT
Ve1[AAK58682.1]     S..LH.....V....D.QI.....E..SK.HF..FVQ..Q....STV.EYF.N............N.Q..

                           290       300       310       320       330       340       350
                    ....|....|....|....|....|....|....|....|....|....|....|....|....|....|
Mlong585_04055      FPDKIFQVKTLQNLELSNNEQLNGTIPELPVSSSLRTMVLSYTNFSGSLPHSIGNLRMLSRIDLSNCNFT
Ve1[AAK58682.1]     ..ER....SV.ES.D..I.KL.R.S..IFFRNG...RIS...........E..S.HQN...LE......Y

                           360       370       380       390       400       410       420
                    ....|....|....|....|....|....|....|....|....|....|....|....|....|....|
Mlong585_04055      GSIPASISNLTELVHLDFSLNSFMGSIHPFSMSKKLVYLDLSRNSLTGSLSSQHFEGLSNLVYINLGVNS
Ve1[AAK58682.1]     ....STMA..RN.GY....F.N.T...PY.RL....T.......G...L..RA......E..H....N.L

                           430       440       450       460       470       480       490
                    ....|....|....|....|....|....|....|....|....|....|....|....|....|....|
Mlong585_04055      LNGTIPPSLFGLPSLQSLQLSNNRFSGQIGEFPIPNPSTLDTLDLSSNRLDGSIPESFFKLEGLNVLSLS
Ve1[AAK58682.1]     .S.SL.AYI.E.....Q.F.YR.Q.V..VD..RNASS.P...V..TN.H.N....K.M.EI.R.K.....

                           500       510       520       530       540       550       560
                    ....|....|....|....|....|....|....|....|....|....|....|....|....|....|
Mlong585_04055      SNLFHGTLKLEKVQSFPNLTRLELGYNKLSVDTS--NMSSFQYPQLSRLNLASCNLLDFPNLRNQSRLLF
Ve1[AAK58682.1]     ..F.R..VP.DLIGRLS..S....S..N.T..A.SS.ST..TF...NI.K....R.QK..D.K...WMMH

                           570       580       590       600       610       620       630
                    ....|....|....|....|....|....|....|....|....|....|....|....|....|....|
Mlong585_04055      LDLSHNSIKGELPSWIWNIGNGSLNHLNLSRNYLVDLQKPFTIPPFLGVLDLNTNSLKGEFPLPPPSAIY
Ve1[AAK58682.1]     ....D.Q.L.AI.N...G..G.G.T.....F.Q.EYVEQ.Y.ASSN.V....HS.R...DLLI..CT...

                           640       650       660       670       680       690       700
                    ....|....|....|....|....|....|....|....|....|....|....|....|....|....|
Mlong585_04055      VDYSNNSFQQAIP-----------VNVANNSLTGTIPESLCDATYLQVLDLSDNNLSGTIPDCLVKG-F
Ve1[AAK58682.1]     ....S.NLNNS..TDIGKSLGFASFFS....GI..I....I.NCS......F.N.A......P..LEYST

                           710       720       730       740       750       760       770
                    ....|....|....|....|....|....|....|....|....|....|....|....|....|....|
Mlong585_04055      TLAVLNLRRNNIEGNIPDVFSSTCGLKTLDLSQNNIGGKIPLSMANCTSLEILNVGNNKIEDVFPCRLKS
Ve1[AAK58682.1]     K.G....GN.KLN.V...S..IG.A.Q.....A..LQ.RL.K.IV..KL..V......RLV.H...M.RN

                           780       790       800       810       820       830       840
                    ....|....|....|....|....|....|....|....|....|....|....|....|....|....|
Mlong585_04055      SSSLRVLVLRSNKFYGNISCTEINGTWPNLQIIDIAFNSFGGKINPRCIATWKGMMLGNGTQLRG-DHIR
Ve1[AAK58682.1]     .N..............LM.DVTRNS.Q........S.N.T.VL.AEFFSN.R...VADDYVET.RN..Q

                           850       860       870       880       890       900       910
                    ....|....|....|....|....|....|....|....|....|....|....|....|....|....|
Mlong585_04055      FEFLKL--------MTVTIKGLELELVKILTVFTAIDFSCNKFSGNIPPTVGDLSSLYILNLSHNSFSGA
Ve1[AAK58682.1]     Y...Q.SKLYYQDTV.L....M........R...S....S.R.Q.A..DAI.N.....V......ALE.P

                           920       930       940       950       960       970       980
                    ....|....|....|....|....|....|....|....|....|....|....|....|....|....|
Mlong585_04055      IPRSIGNLKQLGSLDLSVNQLTGMVPQELTSLNFLSFLNLSYNMLVGKIPDGNQLQTFSALSYIGN---V
Ve1[AAK58682.1]     ..K...K.QM.E.....T.H.S.EI.S..A..T..AA....F.K.F....ST..F.....D.FE..SGLC

                           990      1000      1010      1020      1030      1040      1050
                    ....|....|....|....|....|....|....|....|....|....|....|....|....|....|
Mlong585_04055      GYPAKPTKRWN--------------SIDFNWQFVFTGLGFGVGASLILAPLAFCKEWREKCDEQTDQFMK
Ve1[AAK58682.1]     .L.LNNSCQS.GSASESLPPPTPLPDS.DE.E.I.AAV.YI...ANTISVVW.Y.PVKKWF.KHMEKCLL

                          1060
                    ....|....|....|.
Mlong585_04055      MIYPKYGFSYVRHDSK
Ve1[AAK58682.1]     WFSR.-----------
```

```
                            10        20        30        40        50        60        70
                    ....|....|....|....|....|....|....|....|....|....|....|....|....|....|
Mlong585_04055      MRIQLFSRLLFIS-LIQILI---LVLVSSQCLEDQRSSLLQLKNTLEFNSNSSSKLVKWNQ-SDECCRWE
Ve2 [AAK58012.1]    ..FLH.LWIF..IPFL...LGNEIL......D..K.L.....GSFQYD.TL.N..AR..HNTS...N.N

                            80        90       100       110       120       130       140
                    ....|....|....|....|....|....|....|....|....|....|....|....|....|....|
Mlong585_04055      GVDCDGEGRVIGLNLENETITGGINNSSPLFELQYLKRLNLAFNFFNGAQIPKGIQNLTNLAHLNLSNAG
Ve2 [AAK58012.1]    ..T..LS.H..A.E.DD.K.SS..E.A.A..S....E.....Y.K..VG-..V..G.....TY.......

                           150       160       170       180       190       200       210
                    ....|....|....|....|....|....|....|....|....|....|....|....|....|....|
Mlong585_04055      FGGQVPVEISRLSSLLILDLSTLFGG-PQPLRLENPNLQRLVQNLPRLRELYLDYVNISSQRSEWCQALS
Ve2 [AAK58012.1]    .V..I.MML...TR.VT.......PDFA...K......SHFIE.STE.......G.DL.A..T....S..

                           220       230       240       250       260       270       280
                    ....|....|....|....|....|....|....|....|....|....|....|....|....|....|
Mlong585_04055      SSLPNLTTLSLRSCGLSGPLDSSLGDLRSLSILRLDRNNLSTMIPVFMADFSNLTTLTLGSCFLLGTFPD
Ve2 [AAK58012.1]    .Y.....V....T.RI...I.E..SK.HF..FI...Q.....TV.EYF.N.........S..N.Q....K

                           290       300       310       320       330       340       350
                    ....|....|....|....|....|....|....|....|....|....|....|....|....|....|
Mlong585_04055      KIFQVKTLQNLELSNNEQLNGTIPELPVSSSLRTMVLSYTNFSGSLPHSIGNLRMLSRIDLSNCNFTGSI
Ve2 [AAK58012.1]    R....PV.EF.D..T.KL.S.S..IF.QIG....IS....K......DT.S..QN...LE......SEP.

                           360       370       380       390       400       410       420
                    ....|....|....|....|....|....|....|....|....|....|....|....|....|....|
Mlong585_04055      PASISNLTELVHLDFSLNSFMGSIHPFSMSKKLVYLDLSRNSLTGSLSSQHFEGLSNLVYINLGVNSLNG
Ve2 [AAK58012.1]    .STMA...N..Y....F.N.T..LPY.QGA...I.......G...L..RA......E.......N.....

                           430       440       450       460       470       480       490
                    ....|....|....|....|....|....|....|....|....|....|....|....|....|....|
Mlong585_04055      TIPPSLFGLPSLQSLQLSNNRFSGQIGEFPIPNPSTLDTLDLSSNRLDGSIPESFFKLEGLNVLSLSSNL
Ve2 [AAK58012.1]    SL.AYI.E....KQ.F.YS.Q.V..VD..RNASS.P...V..RN.H.N....K.M.EVGR.K.......F

                           500       510       520       530       540       550       560
                    ....|....|....|....|....|....|....|....|....|....|....|....|....|....|
Mlong585_04055      FHGTLKLEKVQSFPNLTRLELGYNKLSVDTS--NMSSFQYPQLSRLNLASCNLLDFPNLRNQSRLLFLDL
Ve2 [AAK58012.1]    .R..VP.DLIGRLS..S....S..N.T..A.SS.ST..TF...NI.K....R.QK..D.K....MMH...

                           570       580       590       600       610       620       630
                    ....|....|....|....|....|....|....|....|....|....|....|....|....|....|
Mlong585_04055      SHNSIKGELPSWIWNIGNGSLNHLNLSRNYLVDLQKPFTIPPFLGVLDLNTNSLKGEFPLPPPSAIYVDY
Ve2 [AAK58012.1]    .D.Q.L.AI.N...G..G.G.A.....F.Q.EYVEQ.Y.VSSN.V....HS.R...DLLI..ST......

                           640       650       660       670       680       690       700
                    ....|....|....|....|....|....|....|....|....|....|....|....|....|....|
Mlong585_04055      SNNSFQQAIP-----------VNVANNSLTGTIPESLCDATYLQVLDLSDNNLSGTIPDCLVKG-FTLA
Ve2 [AAK58012.1]    .S.NLNNS..TDIGRSLGFASFFS.....I..I....I.NVS......F.N.A......P..LEYSPK.G

                           710       720       730       740       750       760       770
                    ....|....|....|....|....|....|....|....|....|....|....|....|....|....|
Mlong585_04055      VLNLRRNNIEGNIPDVFSSTCGLKTLDLSQNNIGGKIPLSMANCTSLEILNVGNNKIEDVFPCRLKSSSS
Ve2 [AAK58012.1]    ....GN.RLH.V...S.PIG.A.I.....R.IFE..L.K.LV...L..V......SLV.R...M.RN.T.

                           780       790       800       810       820       830       840
                    ....|....|....|....|....|....|....|....|....|....|....|....|....|....|
Mlong585_04055      LRVLVLRSNKFYGNISCTEINGTWPNLQIIDIAFNSFGGKINPRCIATWKGMMLGNGTQLRG-DHIRFEF
Ve2 [AAK58012.1]    .K.........N..LT.NITKHS.K........S.N.T.ML.AE.FTN.R...VAKDYVET.RN..QY..

                           850       860       870       880       890       900       910
                    ....|....|....|....|....|....|....|....|....|....|....|....|....|....|
Mlong585_04055      LKL--------MTVTIKGLELELVKILTVFTAIDFSCNKFSGNIPPTVGDLSSLYILNLSHNSFSGAIPR
Ve2 [AAK58012.1]    .Q.SNLYYQDTV.LI...M........R...S....S.R.Q.K..D.........V......ALE.P..K

                           920       930       940       950       960       970       980
                    ....|....|....|....|....|....|....|....|....|....|....|....|....|....|
Mlong585_04055      SIGNLKQLGSLDLSVNQLTGMVPQELTSLNFLSFLNLSYNMLVGKIPDGNQLQTFSALSYIGN---VGYP
Ve2 [AAK58012.1]    ...K.QM.E.....R.H.S.EI.S..S..T..AV....F.N.F....QS..FE....E.FE..RGLC.L.

                           990      1000      1010      1020      1030      1040      1050
                    ....|....|....|....|....|....|....|....|....|....|....|....|....|....|
Mlong585_04055      AK------------PTKRWNSIDFNWQFVFTGLGFGVGASLILAPLAFCKEWREKCDEQTDQFMKMIYPK
Ve2 [AAK58012.1]    LN-VICKSDTSELK.APSSQDDSYD...I...V.Y....AISI...L.Y.QGNKYF.KHLERML.LMF.R

                          1060      1070      1080      1090      1100      1110      1120
                    ....|....|....|....|....|....|....|....|....|....|....|....|....|....|
Mlong585_04055      YGFSYVRHDSK-----------------------------------------------------------
Ve2 [AAK58012.1]    .W...T.F.PGKVVAVEHYEDETPDDTEDDDEGGKEASLGRYCVFCSKLDFQKNEAMHDPKCTCHMSSSP

                          1130      1140
                    ....|....|....|....|.
Mlong585_04055      ---------------------
Ve2 [AAK58012.1]    NSFPPTPSFFSPLLVIYHKKF
```

C.

```
                             10        20        30        40        50        60        70
                    ....|....|....|....|....|....|....|....|....|....|....|....|....|....|
Mlong585_04031      ---MRIRGLPWFLFIS-LIQILF---LAGVYTQCLDDQRFILLQLKSALAFDLTSSTKL-----------
Ve1[AAK58682.1]     MKM.ATLYF.MV.L.P-SF...SGYHIFL.SS......KSL...F.GS.QY.S.L.K..AKWNDMTSECC

                             80        90       100       110       120       130       140
                    ....|....|....|....|....|....|....|....|....|....|....|....|....|....|
Mlong585_04031      -WNGVDCDGAGHVISLNLESETISGGIENSSTLFRLQYLEELNLAFNFFNSDQIPKGFQNLTSLSYLNLS
Ve1[AAK58682.1]     N....T.NLF....A.E.DD....S......A..S.....S....D.M..VG-..V.ID...N.K.....

                            150       160       170       180       190       200       210
                    ....|....|....|....|....|....|....|....|....|....|....|....|....|....|
Mlong585_04031      NAGFAEQVPIEVSMMSSLIVLDLSTLFGG-AQPLKLEKPNLQQLAQNLTGLRELYLDNVNISSQRSEWGP
Ve1[AAK58682.1]     ....VG.I..TL.RLTR.VT.....ILPFFD......N...SHFIE.S.E.......G.DL....T..CQ

                            220       230       240       250       260       270       280
                    ....|....|....|....|....|....|....|....|....|....|....|....|....|....|
Mlong585_04031      ALSSSLPNLTTLSLRSCGLSGPLDSSLAGFHSLSVLRLDDNNLSATVPEFMSGFSNLTALNLGNCFLQGT
Ve1[AAK58682.1]     S..LH.....V....D.QI.....E..SKL.F..FVQ..Q....S....YFAN.....T.T..S.N....

                            290       300       310       320       330       340       350
                    ....|....|....|....|....|....|....|....|....|....|....|....|....|....|
Mlong585_04031      FPDKIFQLHTLQVLDLSNNKNLNGAIPEFPVSSSLRTMILSYTNFSGWLPNSIGNLRMLSKIDFSNCNFT
Ve1[AAK58682.1]     ..ER...VSV.ES....I..L.R.S..I.FRNG...RIS........S..E..S.HQN..RLEL.....Y

                            360       370       380       390       400       410       420
                    ....|....|....|....|....|....|....|....|....|....|....|....|....|....|
Mlong585_04031      GSIPASVSNLTELVHLDFSLNDFMGSIPPFSMSKKLAYLDLSRNSITGSLSSKHFEGLSNLVYINLGVNS
Ve1[AAK58682.1]     ....STMA..RN.GY....F.N.T....Y.RL....T.......GL..L..RA......E..H....N.L

                            430       440       450       460       470       480       490
                    ....|....|....|....|....|....|....|....|....|....|....|....|....|....|
Mlong585_04031      LNGTIPPSLFDLPSLQRLHLFNNKFSGQITEFPIPNPSSLDTVDLSNNRLEGSIPESFFKLEGLSILSLS
Ve1[AAK58682.1]     .S.SL.AYI.E.....Q.F.YR.Q.V..VD..RNASS.P......T..H.N....K.M.EI.R.KV....

                            500       510       520       530       540       550       560
                    ....|....|....|....|....|....|....|....|....|....|....|....|....|....|
Mlong585_04031      SNLFNGSLKLEKVQRFPNLTRLELGYNRLSVDTS--NMSSFQYPQLSRLNLASCNLLDFPNLRNQSRLLF
Ve1[AAK58682.1]     ..F.R.TVP.DLIG.LS..S....S..N.T..A.SS.ST..TF...NI.K....R.QK..D.K...WMMH

                            570       580       590       600       610       620       630
                    ....|....|....|....|....|....|....|....|....|....|....|....|....|....|
Mlong585_04031      LDLSHNSIKGELPSWIWNIGNGSLNHLNVSRNYLVDLQKPFTIPPFLGVLDLNTNSLKGEFPLPPPSATY
Ve1[AAK58682.1]     ....D.Q.L.AI.N...G..G.G.T...L.F.Q.EYVEQ.Y.ASSN.V....HS.R...DLLI..CT.I.

                            640       650       660       670       680       690       700
                    ....|....|....|....|....|....|....|....|....|....|....|....|....|....|
Mlong585_04031      VDYSNNSFQQGIP-----------VNVANNSLTGTIPESLCDATYLQVLDLSDNNLSGTIPDCLVKG-F
Ve1[AAK58682.1]     ....S.NLNNS..TDIGKSLGFASFFS....GI..I....I.NCS......F.N.A......P..LEYST

                            710       720       730       740       750       760       770
                    ....|....|....|....|....|....|....|....|....|....|....|....|....|....|
Mlong585_04031      TLAVLNLRRNNIEGNIPDVFSSTCGLKTLDLSQNNIGGKIPLSMANCTSLEVLNIGNNKIEDEFPCRLKS
Ve1[AAK58682.1]     K.G....GN.KLN.V...S..IG.A.Q.....A..LQ.RL.K.IV..KL.....V...RLV.H...M.RN

                            780       790       800       810       820       830       840
                    ....|....|....|....|....|....|....|....|....|....|....|....|....|....|
Mlong585_04031      SSSLRVLVLRSNKLYGDISCTEINGSWPNLQIIDIAFNSFGGEINPRCIASWKGMMLGNGTQLRG-EHIR
Ve1[AAK58682.1]     .N...........F..NLM.DVTRN..Q........S.N.T.VL.AEFFSN.R...VADDYVET.RN..Q

                            850       860       870       880       890       900       910
                    ....|....|....|....|....|....|....|....|....|....|....|....|....|....|
Mlong585_04031      FEFLTK--FYYQDAVTVTIKGLELELVKILTLFTAIDLSCNNFSGNIPQTIGDLSSLYVLNLSHNAFSGA
Ve1[AAK58682.1]     Y...QLSKL....T..L....M........RV..S..F.S.R.Q.A..DA..N.............LE.P

                            920       930       940       950       960       970       980
                    ....|....|....|....|....|....|....|....|....|....|....|....|....|....|
Mlong585_04031      IPSSIGNLKQLGSLDLSVNQLTGMVPQELTSLNFLSFLNLSYNMLVGKIPDGNQLQTFSALSYTGNLGLC
Ve1[AAK58682.1]     ..K...K.QM.E.....T.H.S.EI.S..A..T..AA....F.K.F....ST..F.....D.FE..S...

                            990      1000      1010      1020      1030      1040      1050
                    ....|....|....|....|....|....|....|....|....|....|....|....|....|....|
Mlong585_04031      GFPLMNTICYHNSPVGNSP--ASLNKRVYDWQLIIT----------------------------------
Ve1[AAK58682.1]     .L..N.SCQSNG.ASESL.PPTP.PDSDDE.EF.FAAVGYIVGAANTISVVWFYKPVKKWFDKHMEKCLL

                    ....|
Mlong585_04031      -----
Ve1[AAK58682.1]     WFSRK
```

```
                             10        20        30        40        50        60        70
                    ....|....|....|....|....|....|....|....|....|....|....|....|....|....|
Mlong585_04031      MRIRGLPWFLFIS-LIQILF---LAGVYTQCLDDQRFILLQLKSALAFDLTSSTKL------------WN
Ve2 [AAK58012.1]   ..FLHFL.IF..IPFL...LGNEILL.SS......KSL.....GSFQY.S.L.N..ARWNHNTSECCN..

                             80        90       100       110       120       130       140
                    ....|....|....|....|....|....|....|....|....|....|....|....|....|....|
Mlong585_04031      GVDCDGAGHVISLNLESETISGGIENSSTLFRLQYLEELNLAFNFFNSDQIPKGFQNLTSLSYLNLSNAG
Ve2 [AAK58012.1]   ..T..LS....A.E.DD.K..S....A.A..S.....R....Y.K..VG-..V.IG...N.T........

                            150       160       170       180       190       200       210
                    ....|....|....|....|....|....|....|....|....|....|....|....|....|....|
Mlong585_04031      FAEQVPIEVSMMSSLIVLDLSTLFGG-AQPLKLEKPNLQQLAQNLTGLRELYLDNVNISSQRSEWGPALS
Ve2 [AAK58012.1]   .VG.I.MML.RLTR.VT.......PDF.......N...SHFIE.S.E.......G.DL.A..T..CQS..

                            220       230       240       250       260       270       280
                    ....|....|....|....|....|....|....|....|....|....|....|....|....|....|
Mlong585_04031      SSLPNLTTLSLRSCGLSGPLDSSLAGFHSLSVLRLDDNNLSATVPEFMSGFSNLTALNLGNCFLQGTFPD
Ve2 [AAK58012.1]   .Y.....V....T.RI...I.E..SKL.F..FI...Q....T....YFAN.....T.T.SS.N......K

                            290       300       310       320       330       340       350
                    ....|....|....|....|....|....|....|....|....|....|....|....|....|....|
Mlong585_04031      KIFQLHTLQVLDLSNNKNLNGAIPEFPVSSSLRTMILSYTNFSGWLPNSIGNLRMLSKIDFSNCNFTGSI
Ve2 [AAK58012.1]   R...VPV.EF....T..L.S.S..I..QIG....IS....K...S..DT.S..QN..RLEL.....SEP.

                            360       370       380       390       400       410       420
                    ....|....|....|....|....|....|....|....|....|....|....|....|....|....|
Mlong585_04031      PASVSNLTELVHLDFSLNDFMGSIPPFSMSKKLAYLDLSRNSITGSLSSKHFEGLSNLVYINLGVNSLNG
Ve2 [AAK58012.1]   .STMA...N..Y....F.N.T..L.Y.QGA...I.......GL..L..RA......E.......N.....

                            430       440       450       460       470       480       490
                    ....|....|....|....|....|....|....|....|....|....|....|....|....|....|
Mlong585_04031      TIPPSLFDLPSLQRLHLFNNKFSGQITEFPIPNPSSLDTVDLSNNRLEGSIPESFFKLEGLSILSLSSNL
Ve2 [AAK58012.1]   SL.AYI.E....KQ.F.YS.Q.V..VD..RNASS.P......R..H.N....K.M.EVGR.KV......F

                            500       510       520       530       540       550       560
                    ....|....|....|....|....|....|....|....|....|....|....|....|....|....|
Mlong585_04031      FNGSLKLEKVQRFPNLTRLELGYNRLSVDTS--NMSSFQYPQLSRLNLASCNLLDFPNLRNQSRLLFLDL
Ve2 [AAK58012.1]   .R.TVP.DLIG.LS..S....S..N.T..A.SS.ST..TF...NI.K....R.QK..D.K....MMH...

                            570       580       590       600       610       620       630
                    ....|....|....|....|....|....|....|....|....|....|....|....|....|....|
Mlong585_04031      SHNSIKGELPSWIWNIGNGSLNHLNVSRNYLVDLQKPFTIPPFLGVLDLNTNSLKGEFPLPPPSATYVDY
Ve2 [AAK58012.1]   .D.Q.L.AI.N...G..G.G.A...L.F.Q.EYVEQ.Y.VSSN.V....HS.R...DLLI..ST.I....

                            640       650       660       670       680       690       700
                    ....|....|....|....|....|....|....|....|....|....|....|....|....|....|
Mlong585_04031      SNNSFQQGIP-----------VNVANNSLTGTIPESLCDATYLQVLDLSDNNLSGTIPDCLVKG-FTLA
Ve2 [AAK58012.1]   .S.NLNNS..TDIGRSLGFASFFS.....I..I....I.NVS......F.N.A......P..LEYSPK.G

                            710       720       730       740       750       760       770
                    ....|....|....|....|....|....|....|....|....|....|....|....|....|....|
Mlong585_04031      VLNLRRNNIEGNIPDVFSSTCGLKTLDLSQNNIGGKIPLSMANCTSLEVLNIGNNKIEDEFPCRLKSSSS
Ve2 [AAK58012.1]   ....GN.RLH.V...S.PIG.A.I.....R.IFE..L.K.LV...L.....V...SLV.R...M.RN.T.

                            780       790       800       810       820       830       840
                    ....|....|....|....|....|....|....|....|....|....|....|....|....|....|
Mlong585_04031      LRVLVLRSNKLYGDISCTEINGSWPNLQIIDIAFNSFGGEINPRCIASWKGMMLGNGTQLRG-EHIRFEF
Ve2 [AAK58012.1]   .K........FN.NLT.NITKH..K........S.N.T.ML.AE.FTN.R...VAKDYVET.RN..QY..

                            850       860       870       880       890       900       910
                    ....|....|....|....|....|....|....|....|....|....|....|....|....|....|
Mlong585_04031      LTK--FYYQDAVTVTIKGLELELVKILTLFTAIDLSCNNFSGNIPQTIGDLSSLYVLNLSHNAFSGAIPS
Ve2 [AAK58012.1]   .QLSNL....T..LI...M........RV..S..F.S.R.Q.K..D.V..............LE.P..K

                            920       930       940       950       960       970       980
                    ....|....|....|....|....|....|....|....|....|....|....|....|....|....|
Mlong585_04031      SIGNLKQLGSLDLSVNQLTGMVPQELTSLNFLSFLNLSYNMLVGKIPDGNQLQTFSALSYTGNLGLCGFP
Ve2 [AAK58012.1]   ...K.QM.E.....R.H.S.EI.S..S..T..AV....F.N.F....QS..FE....E.FE..R....L.

                            990      1000      1010      1020      1030      1040      1050
                    ....|....|....|....|....|....|....|....|....|....|....|....|....|....|
Mlong585_04031      LMNTICYHNSPVGNSPASLNKRVYDWQLIIT---------------------------------------
Ve2 [AAK58012.1]   .N-V..KSDTSELKPAP.SQDDS....F.F.GVGYGVGAAISIAPLLFYKQGNKYFDKHLERMLKLMFPR

                           1060      1070      1080      1090      1100      1110      1120
                    ....|....|....|....|....|....|....|....|....|....|....|....|....|....|
Mlong585_04031      ----------------------------------------------------------------------
Ve2 [AAK58012.1]   YWFSYTRFDPGKVVAVEHYEDETPDDTEDDDEGGKEASLGRYCVFCSKLDFQKNEAMHDPKCTCHMSSSP

                           1130      1140
                    ....|....|....|....|.
Mlong585_04031      ---------------------
Ve2 [AAK58012.1]   NSFPPTPSFFSPLLVIYHKKF
```
