# Supplementary material for: Chromosome-level genome assembly of Mentha longifolia L. reveals gene organization underlying disease resistance and essential oil traits
Source: G3 (Bethesda). 2022 May 12;12(8):jkac112. doi: 10.1093/g3journal/jkac112 (PMC9339296; doi:10.1093/g3journal/jkac112)

ISPD-L1
Isopiperitenol Dehydrogenase

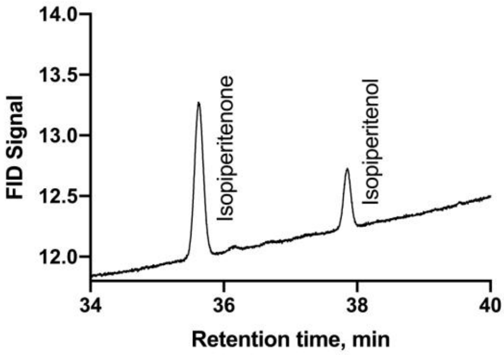

ISPR-1
Isopiperitenone Reductase

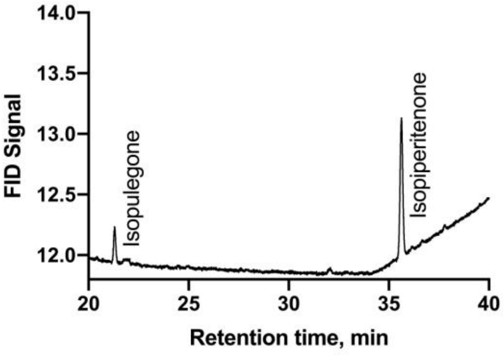

PulR-1
Pulegone Reductase

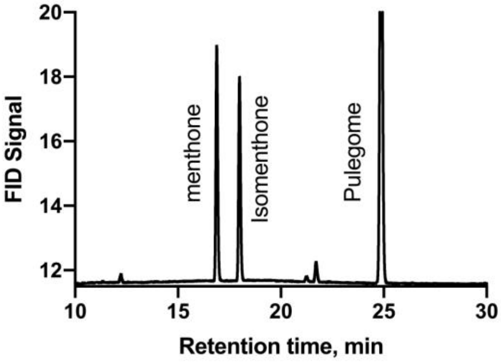

PulR-2
Pulegone Reductase

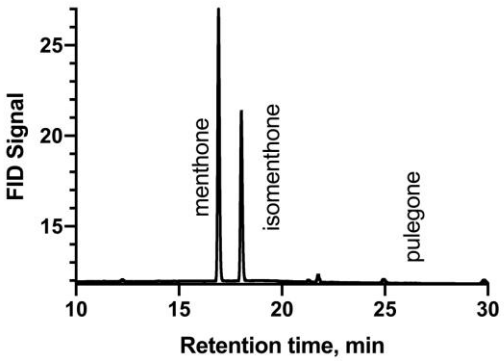

PulR-L1
Pulegone Reductase-Like
(inactive with (+)-pulegone)

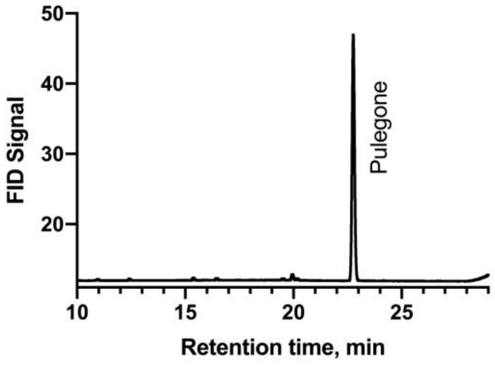

Supplement: jkac112_Figure_S6 [file jkac112_figure_s6.pdf]
